# COVID-19 vaccination induces distinct T-cell responses in pediatric solid organ transplant recipients and immunocompetent children

**DOI:** 10.1038/s41541-024-00866-4

**Published:** 2024-04-05

**Authors:** Katerina Roznik, Jiashu Xue, Georgia Stavrakis, T. Scott Johnston, Divya Kalluri, Rivka Ohsie, Caroline X. Qin, John McAteer, Dorry L. Segev, Douglas Mogul, William A. Werbel, Andrew H. Karaba, Elizabeth A. Thompson, Andrea L. Cox

**Affiliations:** 1 Johns Hopkins Bloomberg School of Public Health, Department of Molecular Microbiology and Immunology, Baltimore, MD USA; 2 Johns Hopkins University School of Medicine, Department of Medicine, Baltimore, MD USA; 3 Johns Hopkins University School of Medicine, Department of Surgery, Baltimore, MD USA; 4 Johns Hopkins University School of Medicine, Department of Pediatrics, Baltimore, MD USA; 5 NYU Grossman School of Medicine, Department of Surgery, New York, NY USA

**Keywords:** RNA vaccines, Paediatric research

## Abstract

Immune responses to COVID-19 vaccination are attenuated in adult solid organ transplant recipients (SOTRs) and additional vaccine doses are recommended for this population. However, whether COVID-19 mRNA vaccine responses are limited in pediatric SOTRs (pSOTRs) compared to immunocompetent children is unknown. Due to SARS-CoV-2 evolution and mutations that evade neutralizing antibodies, T cells may provide important defense in SOTRs who mount poor humoral responses. Therefore, we assessed anti-SARS-CoV-2 IgG titers, surrogate neutralization, and spike (S)-specific T-cell responses to COVID-19 mRNA vaccines in pSOTRs and their healthy siblings (pHCs) before and after the bivalent vaccine dose. Despite immunosuppression, pSOTRs demonstrated humoral responses to both ancestral strain and Omicron subvariants following the primary ancestral strain monovalent mRNA COVID-19 series and multiple booster doses. These responses were not significantly different from those observed in pHCs and significantly higher six months after vaccination than responses in adult SOTRs two weeks post-vaccination. However, pSOTRs mounted limited S-specific CD8^+^ T-cell responses and qualitatively distinct CD4^+^ T-cell responses, primarily producing IL-2 and TNF with less IFN-γ production compared to pHCs. Bivalent vaccination enhanced humoral responses in some pSOTRs but did not shift the CD4^+^ T-cell responses toward increased IFN-γ production. Our findings indicate that S-specific CD4^+^ T cells in pSOTRs have distinct qualities with unknown protective capacity, yet vaccination produces cross-reactive antibodies not significantly different from responses in pHCs. Given altered T-cell responses, additional vaccine doses in pSOTRs to maintain high titer cross-reactive antibodies may be important in ensuring protection against SARS-CoV-2.

## Introduction

Immunosuppressed individuals respond less robustly to vaccination compared to the general population and a two- and three-dose mRNA COVID-19 vaccine regimen demonstrated inadequate immunogenicity in adult solid organ transplant recipients (SOTRs)^1–5^. Although children generally face a lower risk of developing severe COVID-19, those on immunosuppressive regimens are at a higher risk of serious outcomes^6–8^. Members of the International Pediatric Transplant Association reviewed COVID-19 vaccine data in 2022 with focus on pediatric solid organ transplant recipient (pSOTR)-specific issues and concluded that studies of COVID-19 vaccination in pSOTRs are needed to better understand post-vaccine COVID-19 T-cell and antibody kinetics and determine the optimal vaccine schedule^9^. Robust humoral responses against ancestral SARS-CoV-2 variants have been described in healthy children, but there have neither been clinical trials evaluating the immunogenicity of mRNA COVID-19 vaccines in pSOTR populations nor direct comparisons between pSOTRs and healthy children^10^. Observational studies have suggested mRNA vaccination may promote stronger neutralizing responses in pSOTRs compared to adult SOTRs^11–13^. Consequently, a three-dose primary series followed by bivalent boosting has been strongly recommended for adult SOTRs, while guidance on COVID-19 vaccination regimens in pSOTRs has generally been inferred from studies conducted in adults^9,14,15^. While two recent studies evaluated humoral and cellular responses in pSOTRs post-third vaccine dose^16,17^, it remains unknown whether these responses wane by six months following vaccination, as they do in adults. Also, the effect of repeated vaccine dosing and whether the more recently available bivalent vaccines that include Omicron spike (S) sequences improve neutralizing capacity and T-cell responses against evolving variants of concern in pSOTRs remains to be elucidated. Additionally, no studies have comprehensively evaluated phenotypic and functional characteristics of mRNA vaccine-induced S antigen-specific T cells in the pSOTR population, which may provide additional lines of defense despite evasion of neutralizing antibody by the Omicron subvariants^18^.

In this study, we assessed mRNA COVID-19 vaccine-induced humoral and T-cell responses in pSOTRs and their immunocompetent siblings (pediatric healthy controls; pHC) against both the ancestral SARS-CoV-2 strain and the Omicron BA.5 subvariant. Omicron BA.5, initially identified in South Africa in February 2022, rapidly became the prevalent circulating subvariant in the U.S. by July 2022. Given the significant mutations observed in the Omicron BA.5 S protein^19^, our objectives were to compare neutralizing capacity against the ancestral strain and BA.5, to assess whether T cells induced by ancestral strain monovalent vaccination in both pSOTRs and pHCs retain the ability to recognize Omicron subvariants, and to comprehensively evaluate vaccine-induced T-cell responses. Bivalent mRNA COVID-19 vaccines that encode both the ancestral and Omicron BA.4/5 S proteins became available in September 2022 and were approved for children aged 6 months and older^20,21^. Thus, we also evaluated humoral and T-cell responses in pSOTRs who received bivalent mRNA vaccine. Lastly, we investigated whether prior COVID-19 history impacted these measures of immunogenicity.

## Results

### Following on average one additional ancestral mRNA vaccine dose, pediatric solid organ transplant recipients do not exhibit significantly different humoral responses compared to their healthy siblings

**Fig. 1:**
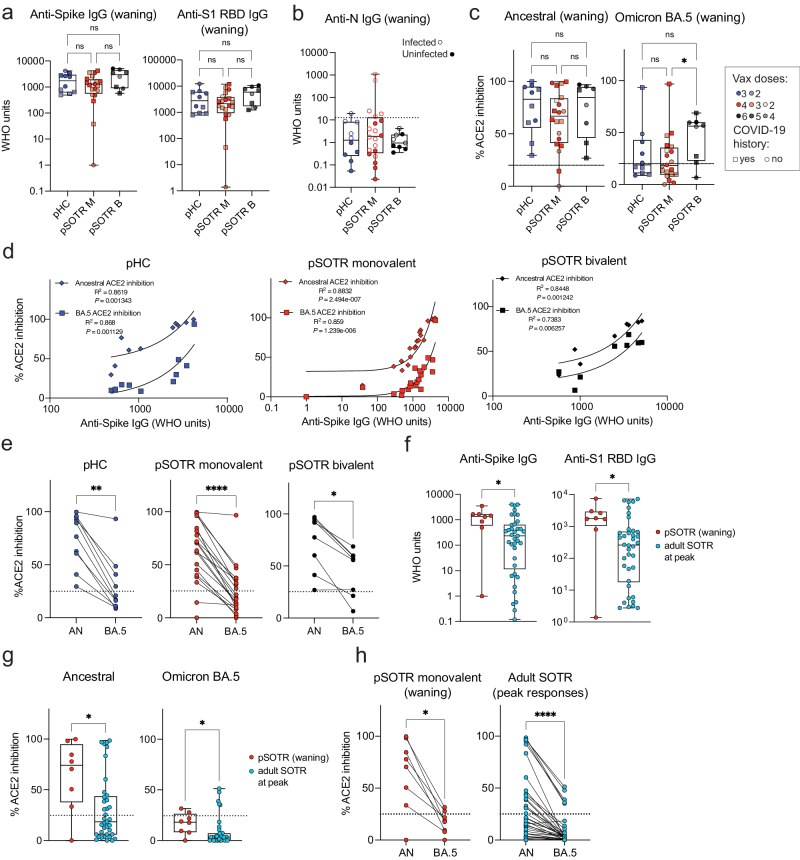
Pediatric solid organ transplant recipients with an extra dose of ancestral monovalent vaccine do not exhibit significantly different humoral responses from their healthy siblings. **a** Anti-S and anti-S1 RBD IgG titers in pHCs (*n* = 10) and pSOTR monovalently vaccinated (M) (*n* = 20) at approximately 6 months (180 days) since last vaccination and pSOTRs who received the bivalent dose (B) (*n* = 8) 300 days post-vaccination. Squares denote individuals with history of COVID-19, circles represent no history of COVID-19. Darker shades of color indicate more vaccines received. Kruskal–Wallis test, ns = not significant. **b** Anti-nucleocapsid IgG titers. The WHO cutoff of 12.3 units (positivity for natural infection) is depicted by dotted line. Filled circles represent individuals with self-reported or documented SARS-CoV-2 infection. **c** Percent ACE2 binding inhibition of ancestral strain and Omicron BA.5. Squares denote individuals with history of COVID-19, circles represent no history of COVID-19. Darker shades of color indicate more vaccines received. Kruskal–Wallis tests, *p < 0.05. The dotted line represents 25% ACE2 inhibition (limit of detection). **d** Correlations between anti-S IgG titers and ACE2 binding inhibition of ancestral strain and Omicron BA.5. **e** Matched pair percent ACE2 binding inhibition of ancestral strain vs. Omicron BA.5 for each individual within the groups. Wilcoxon matched-pairs rank test, *p < 0.05, **p < 0.01, ***p < 0.001, ****p < 0.0001. **f** Anti-S and anti-S1 RBD IgG titers in pSOTR (*n* = 8) six months since last vaccination and adult SOTRs (*n* = 38) at peak vaccine responses (day 14). Mann–Whitney test, *p < 0.05. **g** Percent ACE2 binding inhibition of ancestral strain and Omicron BA.5 in pSOTRs (*n* = 8) and adult SOTRs (*n* = 38). Mann–Whitney tests, *p < 0.05. The dotted line represents 25% ACE2 inhibition (limit of detection). **h** Matched pair percent ACE2 binding inhibition of ancestral strain vs. Omicron BA.5 in pSOTRs (*n* = 8) and adult SOTRs (*n* = 38). Wilcoxon matched-pairs rank test, *p < 0.05, ****p < 0.0001. In (**a**–**c**, **f**, **g**), boxplots were used to summarize data (median, 1st–3rd quartiles (IRQ), whiskers represent minimum and maximum values).

Humoral responses were evaluated in pHCs and pSOTRs who received the ancestral monovalent vaccines only (pSOTR M) after approximately 200 days and pSOTR bivalent recipients (pSOTR B) after 300 days post-vaccination, just before the time at which boosting has been recommended for older adults and adult SOTRs due to waning immunity. The pSOTR M group received, on average one additional mRNA vaccine dose compared to pHCs (median three vs. two doses, respectively), while pSOTR B group received on average five mRNA vaccines, including the bivalent dose (Supplementary Table 1). Anti-S immunoglobulin (Ig)-G and anti-S1 receptor binding domain (RBD) IgG titers were not significantly different between pHCs and pSOTR groups (Fig. 1a). Interestingly, despite a previously documented infection in 11 (55%) pSOTR M, four (44%) pSOTR B, and five (50%) pHC participants, we observed no detectable anti-nucleocapsid (N) IgG in most individuals (Fig. 1b). While failure to acquire anti-N IgG following infection in children has not been reported, this is consistent with what has been observed in adults who were infected following vaccination^22^.

To assess antibody functionality, we measured percent angiotensin-converting enzyme 2 (ACE2) binding inhibition of the SARS-CoV-2 ancestral strain and Omicron BA.5 as a surrogate of neutralizing antibody function. This assay was previously validated in adult SOTRs^23,24^ and strongly correlated with live virus-neutralizing antibody titers in transplant recipients. Inhibition of the ancestral strain and BA.5 following monovalent vaccination was not significantly different between pSOTRs and pHCs; however, the pSOTR B group continued to exhibit slightly enhanced inhibition of Omicron BA.5 ten months post-vaccination (Fig. 1c). Although anti-S IgG titers positively correlated with ACE2 inhibition of both ancestral strain and Omicron BA.5 for all groups, there was a significant decrease in BA.5 surrogate neutralization compared to the ancestral strain, especially in monovalently vaccinated pHCs and pSOTRs (Fig. 1d, e).

Next, we compared antibody responses between pSOTR six months post-vaccination to 38 adult SOTRs two weeks post-third mRNA COVID-19 vaccine dose (i.e., peak response). To minimize heterogeneity, this analysis included only pSOTRs and adult SOTRs who were previously uninfected and who received three mRNA ancestral monovalent doses. pSOTRs six months post-vaccination exhibited significantly higher IgG titers (Fig. 1f) and greater neutralization capacity (Fig. 1g) compared to adult SOTRs at peak vaccine responses. Comparing percent inhibition of ancestral and Omicron BA.5 protein binding to ACE2 in the same individuals demonstrates that pSOTR and adult SOTRs had significantly lower BA.5 surrogate neutralization versus ancestral strain (Fig. 1h). Collectively, these data indicate that an additional mRNA vaccine dose in pSOTRs induced comparable IgG titers and neutralization capacity compared to immunocompetent children and that, despite waning, pSOTRs had significantly enhanced antibody responses six months post-vaccination compared to adult SOTRs at peak vaccine responses. Additionally, bivalent doses enhanced neutralization capacity against Omicron BA.5 in pSOTRs.

### Bivalent boosting improves vaccine-induced antibody responses to both the ancestral and Omicron BA.5 variants

**Fig. 2:**
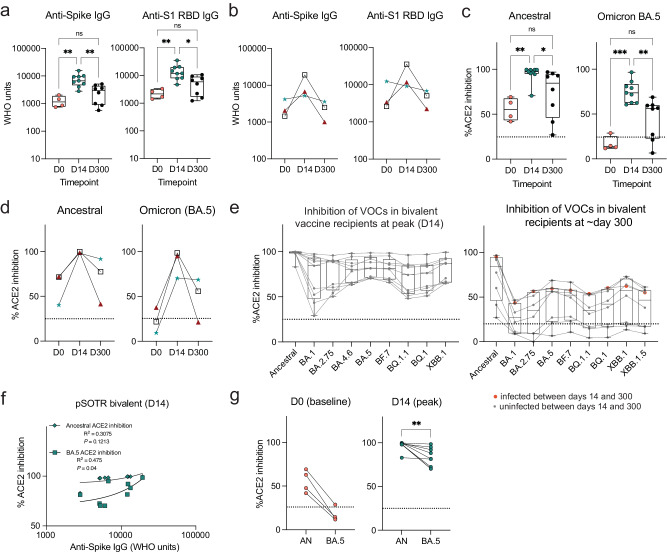
Bivalent boosting improves vaccine-induced antibody responses to both the ancestral strain and Omicron BA.5 and wane over time. **a** Anti-S and anti-S1 RBD IgG titers in the pSOTR bivalent group at days 0 (D0; pre-bivalent) (*n* = 4), 14 (D14; peak responses) (*n* = 9) and 300 (D300; waning responses) (*n* = 8). Kruskal–Wallis test, *p < 0.05, **p < 0.01. Boxplots were used to summarize data (median, 1st–3rd quartiles (IRQ), whiskers represent minimum and maximum values). **b** Anti-S and anti-S1 RBD IgG titers at days 0, 14, and 300 since the bivalent vaccination in three individuals with matched plasma samples. **c** Percent ACE2 binding inhibition of ancestral strain and Omicron BA.5 in bivalent vaccine pSOTR recipients. Kruskal–Wallis tests, *p < 0.05, **p < 0.01, ***p < 0.001. The dotted line represents 25% ACE2 inhibition (limit of detection). Boxplots were used to summarize data (median, 1st–3rd quartiles (IRQ), whiskers represent minimum and maximum values). **d** Percent ACE2 binding inhibition of the ancestral strain and Omicron BA.5 at days 0, 14, and 300 since the bivalent vaccination in three individuals with matched plasma samples. **e** Percent ACE2 binding inhibition of the ancestral strain and Omicron variants of concern (VOC) in bivalent dose recipients at peak (left) and day 300 (right). A single individual was infected between days 14 and 300 (red circles). Boxplots were used to summarize data (median, 1st–3rd quartiles (IRQ), whiskers represent minimum and maximum values). **f** Correlations between anti-S IgG titers and ACE2 binding inhibition of ancestral strain and Omicron BA.5 at peak responses (see Fig. 1d for correlations at day 300). **g** Matched pair percent ACE2 binding inhibition of ancestral strain vs. Omicron BA.5 at days 0 and 14 (see Fig. 1e for day 300). Wilcoxon matched-pairs rank test, **p < 0.01.

As anticipated, anti-S IgG antibody levels in pSOTRs significantly increased after the bivalent vaccination (day 14) compared to pre-vaccination (day 0), followed by a decline in responses approximately ten months later (day 300) (Fig. 2a). Given that monovalent vaccination was discontinued, it was not possible to determine if the same effect would be observed if the additional dose were not a bivalent vaccine. Three individuals in the bivalent group had matched pre- and post-bivalent dose samples. Anti-S IgG titers increased for all three, and anti-S1 RBD IgG titers increased for two out of the three individuals at day 14, before decreasing again at six months post-vaccination (Fig. 2b). Bivalently boosted pSOTRs also displayed a high capacity to neutralize both the ancestral strain and Omicron BA.5 at day 14 (Fig. 2c) in the surrogate neutralization assay. The three pSOTRs with matched pre- and post-bivalent dose samples all demonstrated a significant increase in ACE2 inhibition following the bivalent boost, especially for the BA.5 subvariant (Fig. 2d), including the child who did not demonstrate globally increased antibody titers (Fig. 2b, green star). Additionally, at day 14, bivalent recipients exhibited robust ACE2 binding inhibition of other Omicron subvariants, including BA.1, BA.2.75, BA.4.6, BF.7, and the more recently circulating BQ.1.1, BQ.1, and XBB.1, that was not significantly different from that of BA.5 surrogate neutralization (Fig. 2e). Importantly, no individuals had neutralizing capacity below the 25% ACE2 binding inhibition cutoff (previously shown to be specific for the presence of live-virus neutralization in SOTRs), suggesting overall excellent performance against Omicron subvariants. This suggests that not only are pSOTRs capable of effectively neutralizing Omicron BA.5, but also that an additional dose may help to protect this population from newly emerging SARS-CoV-2 variants. Ten months post-bivalent dose, pSOTRs exhibited decreased neutralizing capacity for every Omicron subvariant tested, with approximately half of individuals staying above the 25% cutoff (Fig. 2e). Lastly, while there was a positive correlation between anti-S IgG titers and ACE2 inhibition for both the ancestral strain and Omicron BA.5, decreased BA.5 inhibition was observed compared to the ancestral strain (Fig. 2f, g). Together, these findings provide evidence that either bivalent boosting or an additional mRNA vaccine dose significantly enhances antibody responses in pSOTRs, including increased total anti-SARS-CoV-2 IgG titers and improved surrogate neutralizing capacity against the ancestral strain, Omicron BA.5 and other variants of concern. However, anti-SARS-CoV-2 IgG titers and neutralizing capacities wane by ten months post-vaccination, suggesting that additional boosting might be beneficial in this population.

### Humoral responses in vaccinated uninfected and vaccinated infected individuals are not significantly different

Although 22% of the U.S. pediatric population has reported a positive COVID-19 test since the beginning of the COVID-19 pandemic, infection seroprevalence could be as high as 96%^21,25^. In this cohort, approximately 50% of participants in each group reported an infection (Supplementary Table 1). Given the frequency of pediatric infection in the U.S. and that little is known about SARS-CoV-2 vaccine immunity in pSOTRs since the Omicron variant emerged, we stratified antibody responses by COVID-19 infection status (Supplementary Table 1).

One individual in the pSOTR group had COVID-19 twice and one of the infections occurred when Omicron BA.5 was circulating. All other study participants were infected between May 2021 and June 2022 (i.e., before Omicron BA.5 became the dominant variant in the U.S.). There were no significant differences in total IgG titers and ancestral or BA.5 surrogate neutralization between vaccinated uninfected and vaccinated infected individuals within each group (Supplementary Fig. 1a, b). This suggests that the antibody results likely reflect vaccine-induced rather than infection-induced or hybrid immune responses.

### S-specific CD4 T^+^ cells in immunocompetent children produce more interferon-γ compared to pediatric solid organ transplant recipients

**Fig. 3:**
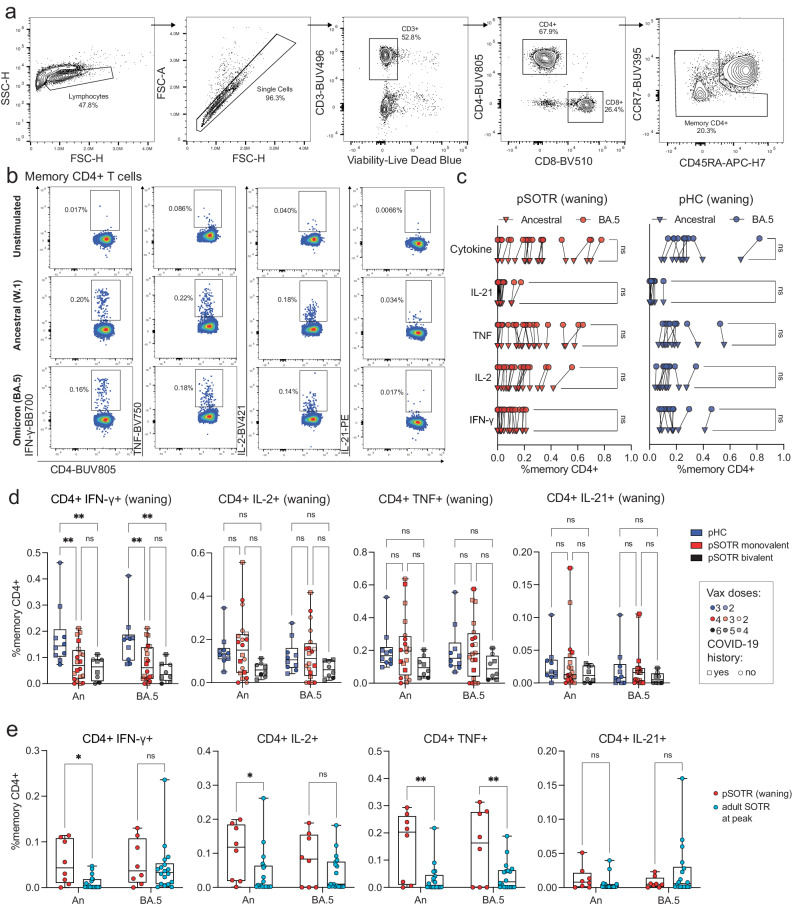
S-specific CD4 T^+^ cells in immunocompetent children produce more interferon-γ compared to pediatric solid organ transplant recipients. **a** Representative flow cytometry gating of cytokine-producing S-specific CD4^+^ T cells. **b** Representative cytokine production by S-specific CD4^+^ T cells unstimulated (baseline) or stimulated with ancestral (W.1) or Omicron BA.4/5 S protein peptides. **c** Cytokine production by S-specific CD4^+^ T cells in responses to ancestral and BA.4/5 S peptides in pSOTRs and pHCs vaccinated with monovalent mRNA COVID-19 vaccines. One-way ANOVA with Tukey correction, ns = not significant. **d** Frequencies of cytokine-producing S-specific CD4^+^ T cells in response to BA.4/5 or ancestral S peptide stimulation. Squares denote individuals with history of COVID-19, circles represent no history of COVID-19. Darker shades of color indicate more vaccines received. Two-way ANOVA with Tukey correction for multiple comparisons, **p < 0.01. An = ancestral strain. Boxplots were used to summarize data (median, 1st–3rd quartiles (IRQ), whiskers represent minimum and maximum values). **e** Frequencies of cytokine-producing S-specific CD4^+^ T cells in response to BA.4/5 or ancestral S peptides in pSOTRs (*n* = 8) six months since the last vaccine dose and adult SOTRs (*n* = 19) for which we had PBMC samples at peak vaccine responses (day 14). Two-way ANOVA with Tukey correction, *p < 0.05, **p < 0.01. An = ancestral strain. Boxplots were used to summarize data (median, 1st–3rd quartiles (IRQ), whiskers represent minimum and maximum values).

To examine S antigen-specific T cells induced by ancestral monovalent vaccination and vaccination plus infection, participant PBMCs were stimulated with overlapping ancestral (W.1) and Omicron BA.4/5 S protein peptides. Subsequently, the production of interferon (IFN)-γ, tumor necrosis factor (TNF), interleukin (IL)-2, and IL-21 cytokines was assessed by flow cytometry (Fig. 3a, b). As with antibody responses, no significant differences in CD4^+^ T-cell responses were observed between uninfected vaccinated participants and those vaccinated and previously infected (Supplementary Fig. 2). In contrast to the antibody responses, pSOTRs and pHCs vaccinated with monovalent mRNA vaccines exhibited no significant differences in the frequency of CD4^+^ T-cell responses recognizing Omicron BA.5 epitopes compared to the ancestral strain (Fig. 3c). Interestingly, despite receiving one fewer vaccine dose on average than the pSOTR M group and more than two fewer doses on average than the pSOTR B group, pHCs demonstrated greater production of IFN-γ by S-specific CD4^+^ T cells in response to both ancestral and BA.4/5 peptides (Fig. 3d). This result remained significant after accounting for prior COVID-19 infection, immunosuppression (mycophenolate mofetil use), liver transplant history, age, number of vaccines received, and time between vaccination and sample collection (Supplementary Table 2). Production of other cytokines was not statistically significantly different between pSOTRs and pHCs six months post ancestral vaccination or pSOTRs ten months post bivalent dose. However, compared to three times vaccinated uninfected adult SOTRs at peak vaccine response^26^, three-times vaccinated and uninfected pSOTRs produced significantly more IFN-γ, IL-2, and TNF in response to ancestral S protein stimulation, and TNF in response to BA.4/5 S protein stimulation six months post-vaccination (Fig. 3e). In sum, while immunocompetent children produced significantly more IFN-γ in response to S peptide stimulation, pSOTRs showed comparable production of all other cytokines and greater production of most cytokines assessed six months post-vaccination compared to adult SOTRs at peak responses.

### CD4^+^ T-cell responses are improved after bivalent boosting and maintain cross-reactivity against Omicron BA.5

**Fig. 4:**
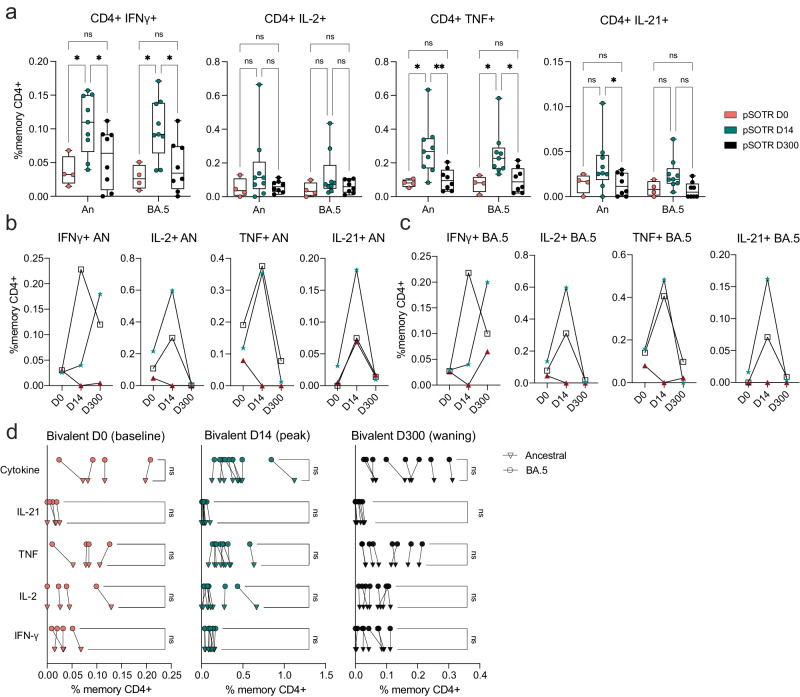
CD4^+^ T-cell responses are improved after bivalent boosting and maintain cross-reactivity against Omicron BA.5. **a** Frequencies of cytokine-producing S-specific CD4^+^ T cells in response to BA.4/5 or ancestral S peptides at 0, 14, and 300 days post-bivalent vaccination. Two-way ANOVA with Tukey correction, *p < 0.05, **p < 0.01. An = ancestral strain. Boxplots were used to summarize data (median, 1st–3rd quartiles (IRQ), whiskers represent minimum and maximum values). **b**, **c** Cytokine production by S-specific CD4^+^ T cells in response to BA.4/5 or ancestral S peptides for three pSOTR individuals with matched PBMC samples at 0, 14, and 300 days post-bivalent vaccination. **d** Cytokine production by S-specific CD4^+^ T cells in responses to ancestral and BA.4/5 S peptides at days 0, 14, and 300 post-bivalent vaccination. One-way ANOVA with Tukey correction, ns = not significant.

Bivalent boosting in pSOTRs enhanced CD4^+^ T-cell production of IFN-γ and TNF following ancestral S peptide stimulation compared to days 0 and 300, and IL-21 compared to day 300 (Fig. 4a). Similarly, bivalent recipients exhibited improved production of IFN-γ and TNF in response to BA.4/5 peptides compared to days 0 and 300 (Fig. 4a). This suggests that memory CD4^+^ T cells in pSOTRs can be boosted and successfully recalled following additional mRNA vaccine doses and/or the bivalent dose, but wane over time to pre-bivalent levels. Two out of three matched bivalent recipients exhibited increased CD4^+^ T-cell responses following bivalent boosting in response to both ancestral and BA.4/5 peptides (Fig. 4b, c). Interestingly, IFN-γ production increased in one individual in response to ancestral peptide, and two individuals in response to BA.4/5 peptides, however, neither had a reported SARS-CoV-2 infection between days 14 and 300 post-vaccination. There were no differences in the frequency of CD4^+^ T-cell responses recognizing ancestral and Omicron BA.4/5 epitopes in pSOTRs bivalent recipients at days 0, 14, and 300 (Fig. 4d). Overall, bivalent boosting in pSOTRs led to an enhanced cytokine production by CD4^+^ T cells at peak with conserved recognition of ancestral strain and BA.4/5 peptides. CD4^+^ T-cell cytokine production waned over time, which is in contrast with cytokine production observed in immunocompetent children after two to three doses of ancestral monovalent vaccines (Fig. 3).

### CD8^+^ T-cell responses are low, including following bivalent boosting

**Fig. 5:**
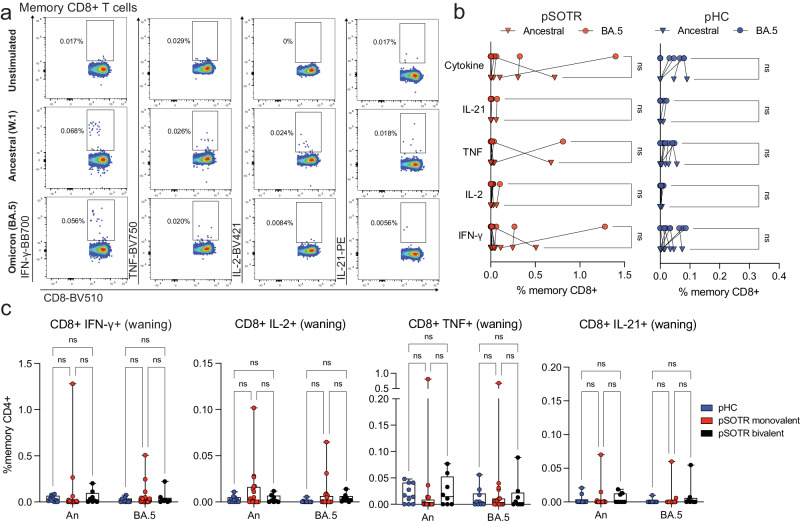
CD8^+^ T-cell responses are low six+ months post-vaccination. **a** Representative cytokine production by S-specific CD8^+^ T cells unstimulated (baseline) or stimulated with ancestral (W.1) or Omicron BA.4/5 S protein peptides. **b** Cytokine production by S-specific CD8^+^ T cells in response to ancestral and BA.4/5 S peptides in monovalently vaccinated pSOTRs and pHCs. One-way ANOVA with Tukey correction, ns = not significant. **c** Frequencies of cytokine-producing S-specific CD8^+^ T cells in response to BA.4/5 or ancestral S peptides. Two-way ANOVA with Tukey correction. An = ancestral strain. Boxplots were used to summarize data (median, 1st–3rd quartiles (IRQ), whiskers represent minimum and maximum values).

**Fig. 6:**
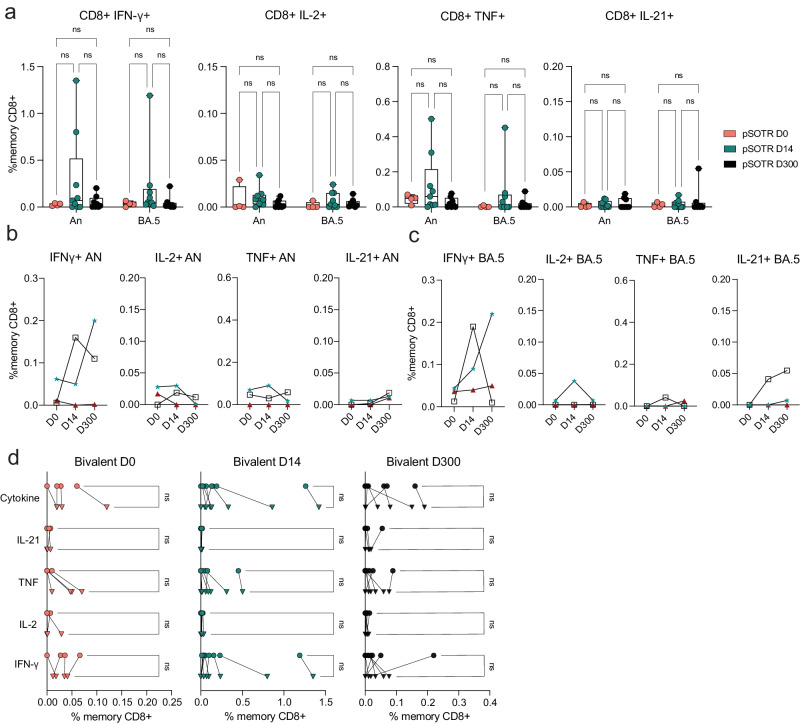
Bivalent boosting does not improve CD8^+^ T-cell responses vaccine responses in pSOTRs. **a** Frequencies of cytokine-producing S-specific CD8^+^ T cells in response to BA.4/5 or ancestral S peptides in pSOTRs at days 0, 14, and 300 post-bivalent dose. Two-way ANOVA with Tukey correction. An = ancestral strain. Boxplots were used to summarize data (median, 1st–3rd quartiles (IRQ), whiskers represent minimum and maximum values). **b**, **c** Cytokine production by S-specific CD8^+^ T cells in response to BA.5 or ancestral S peptides for three pSOTR individuals with matched PBMC samples at days 0, 14, and 300 post-bivalent dose. **d** Cytokine production by S-specific CD8^+^ T cells in response to ancestral and BA.4/5 S peptides in pSOTRs bivalent recipients at days 0, 14, and 300 post-bivalent dose. One-way ANOVA with Tukey correction, ns = not significant.

Live attenuated and viral vector-based vaccines have traditionally elicited strong CD8^+^ T-cell responses, which may offer additional protection independent of antibody responses^27–29^. Although mRNA vaccine-induced S-specific CD8^+^ T-cell responses in pSOTRs have not been comprehensively characterized, studies in adult SOTRs have reported limited CD8^+^ T-cell responses^26,30^. For both pSOTRs and pHCs, the overall frequency of cytokine producing CD8^+^ T cells upon stimulation with ancestral or Omicron BA.4/5 peptides remained low, often comparable to background (S peptide unstimulated) levels (Fig. 5a–c). Furthermore, at six months post bivalent dose, pSOTRs exhibited limited cytokine production by S-specific CD8^+^ T cells (Fig. 5c). Bivalent boosting slightly improved CD8^+^ T-cell production of IFN-γ and TNF at peak vaccine responses, but not significantly (Fig. 6a–d). Overall, cytokine production by CD8^+^ T cells was limited, demonstrating that mRNA COVID-19 vaccines induce more robust CD4^+^ than CD8^+^ T-cell responses in pediatric populations.

### No significant difference in CD8^+^ T-cell responses in vaccinated uninfected and vaccinated and infected individuals

Early studies reported that SARS-CoV-2 infection induces robust CD8^+^ T-cell responses in adults^31,32^. Hence, we stratified CD8^+^ T-cell responses by previous history of COVID-19. Comparable to CD4^+^ T-cell and antibody results, CD8^+^ T-cell responses were not significantly different between those with and without a history of SARS-CoV-2 infection (Supplementary Fig. 3). Additionally, as mentioned previously, all cytokines tested were present in very low frequencies preventing trend evaluation in each group. These results further confirm that the responses we observed were induced mainly by vaccination and that S-specific CD8^+^ T-cell responses were low compared to CD4^+^ T-cell responses.

### pSOTRs produce qualitatively different polyfunctional CD4^+^ T cells compared to pHCs

**Fig. 7:**
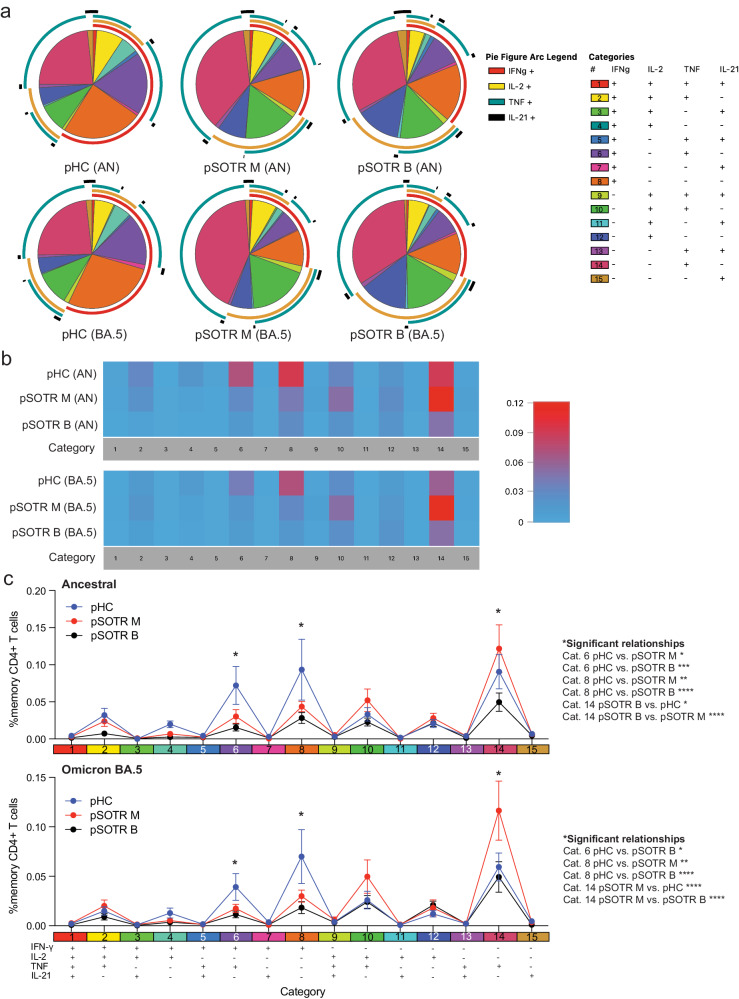
pSOTRs produce qualitatively different polyfunctional CD4^+^ T cells compared to pHCs in response to vaccination. **a** Ancestral and BA.4/5 peptide stimulation induced no significant differences in frequencies of polyfunctional CD4^+^ T cells in each group. Pie charts are broken down by the 15 cytokine combination categories. Arcs identify slices of the pie that express each specific cytokine. **b** Heatmap identifying absolute differences between groups for each category (1–15). **c** Frequencies of CD4^+^ T cells producing cytokine combinations in response to ancestral or BA.4/5 peptides. Two-way ANOVA with Tukey correction, *p < 0.05, **p < 0.01, ***p < 0.001, ****p < 0.0001. Error bars represent standard error of the mean.

Polyfunctionality is defined as the ability of T cells to produce more than one cytokine simultaneously and has been associated with protection in previous studies against other infections^33,34^. Due to overall low CD8^+^ T-cell responses (Figs. 5, 6), we evaluated polyfunctionality in CD4^+^ T cells only. Ancestral and BA.4/5 peptide stimulation induced no significant difference in overall frequencies of S-specific polyfunctional CD4^+^ T cells within each group (Fig. 7a). However, pHC participants demonstrated increased polyfunctionality in response to ancestral and BA.4/5 S peptide stimulation compared to the pSOTR groups (Fig. 7b, c), primarily due to increased simultaneous production of TNF and IFN-γ (category 6, purple). Additionally, S-specific CD4^+^ T cells in pHCs produced significantly more IFN-γ only (category 8, orange), while pSOTR CD4^+^ T cells produced more TNF only in response to both ancestral and BA.4/5 peptides (category 14, dark pink) (Fig. 7c). Together, these findings suggest that while pSOTRs produce polyfunctional CD4^+^ T cells in response to mRNA vaccination, they are qualitatively different compared to polyfunctional CD4^+^ T cells produced by immunocompetent children, and lack robust production of IFN-γ.

### Bivalent boosting improved CD4^+^ T-cell polyfunctionality in pSOTRs

**Fig. 8:**
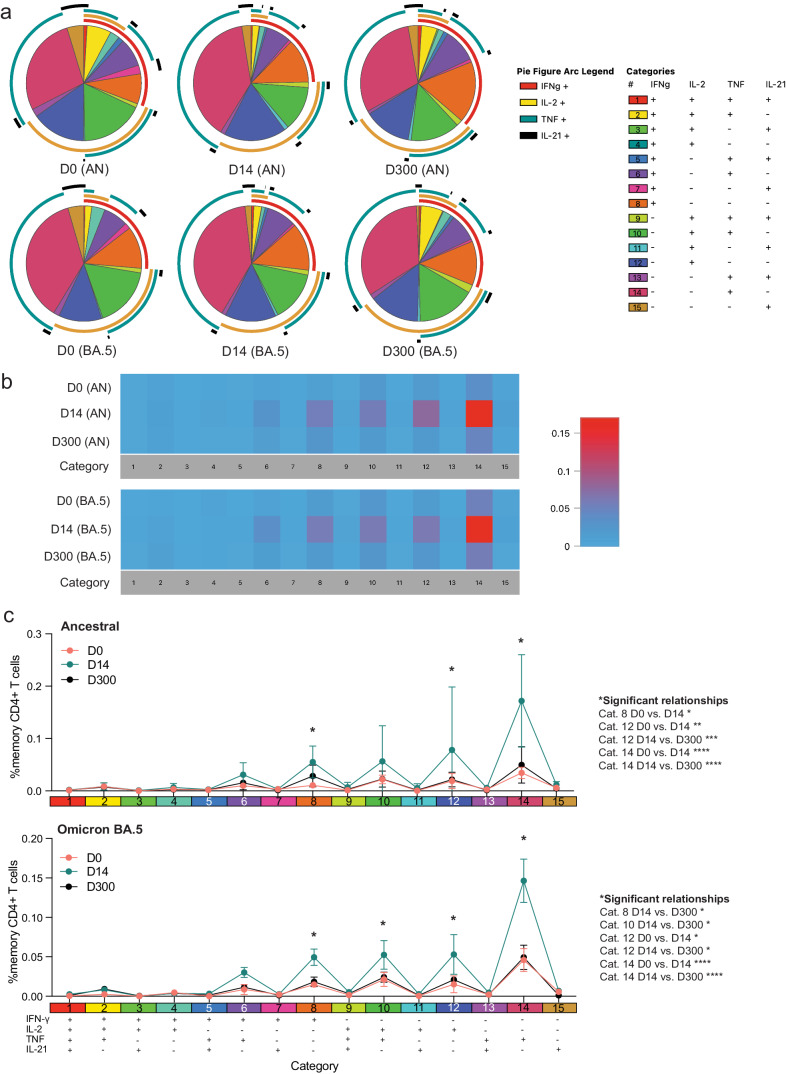
Bivalent boosting temporarily improved CD4^+^ T-cell polyfunctionality in pSOTRs. **a** Cytokine production by CD4^+^ T cells in response to ancestral and Omicron BA.4/5 peptides at days 0, 14, and 300 post-bivalent vaccination. Pie charts depict the 15 cytokine combination categories. Arcs identify slices of the pie that express each specific cytokine. **b** Heatmap identifying absolute differences between groups for each category. **c** Frequencies of CD4^+^ T cells producing cytokine combinations in response to ancestral or BA.4/5 peptide stimulation. Two-way ANOVA with Tukey correction, *p < 0.05, **p < 0.01, ***p < 0.001, ****p < 0.0001. Error bars represent standard error of the mean.

We next assessed S-specific CD4^+^ T-cell polyfunctionality and whether an additional dose of vaccine could increase IFN-γ production by pSOTRs (Fig. 8). We did not observe significant differences in ancestral or BA.4/5-induced cytokine production (Fig. 8a), but overall polyfunctionality was significantly improved shortly after post-bivalent vaccination (Fig. 8b, c). Bivalent boosting primarily increased the frequency of CD4^+^ T cells simultaneously producing IL-2 and TNF (category 10, green) in response to BA.4/5 S peptides at peak responses. Additionally, the production of IFN-γ only (category 8, orange), IL-2 only (category 12, dark blue), and TNF only (category 14, dark pink) significantly increased at peak compared to prior and ten months post-bivalent boosting (Fig. 8b, c). In sum, bivalent boosting enhances IFN-γ production by CD4^+^ T cells 14 days post-vaccination, but not long-term, as seen in pHCs. Instead, bivalent doses in pSOTRs enhanced long-term TNF and IL-2 cytokine production (Figs. 7c, 8c).

Subsequently, we investigated whether CD4^+^ T-cell polyfunctionality differs in the nine pSOTR bivalent recipients depending on history of COVID-19 (Supplementary Fig. 4). While infected individuals tended to exhibit slightly increased production of IFN-γ (category 8) and TNF (category 14), no significant differences in cytokine production or polyfunctionality between individuals with or without previously documented SARS-CoV-2 infection were noted (Supplementary Fig. 4a, b), further indicating that the responses observed in this study are predominantly vaccine-induced.

### pSOTRs generate metabolically active but qualitatively distinct CD4^+^ T cells following mRNA vaccination

**Fig. 9:**
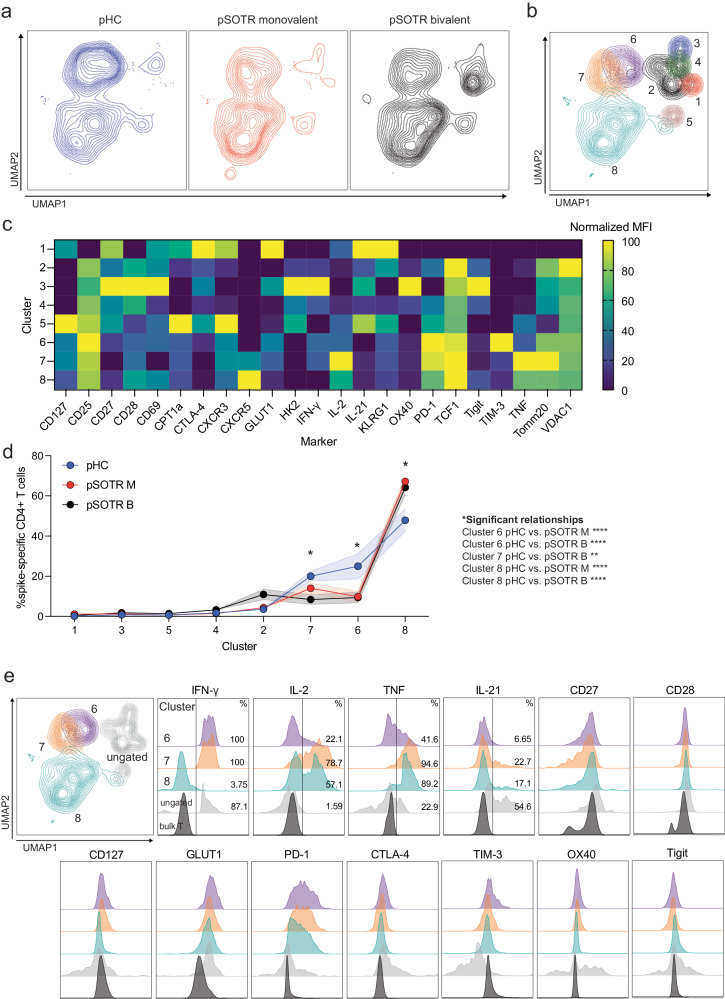
pSOTRs generate metabolically active but qualitatively distinct CD4^+^ T cells following mRNA vaccination compared to pHCs. **a** UMAP dimension reduction plot for each group. **b** Unsupervised clustering algorithm Xshift identified 8 clusters on the UMAP. **c** Heatmap of normalized mean fluorescent intensity (MFI) values of markers expressed in each cluster. **d** Frequency of clusters in each group. Two-way ANOVA with Tukey correction, *p < 0.05, **p < 0.01, ***p < 0.001, ****p < 0.0001. Error bars represent standard error of the mean. **e** MFI plots for significant clusters determined in (**d**), ungated clusters (gray) and bulk T cells (black).

We then comprehensively evaluated phenotypic and functional markers of S-specific CD4^+^ T cells induced in response to ancestral and BA.4/5 peptides using high parameter flow cytometry. The panel includes 29 surface and intracellular markers designed to evaluate T-cell subsets, metabolism, activation, and exhaustion phenotypes (Supplementary Table 3). No significant differences in S-specific CD4^+^ T-cell phenotypes were observed between responses to the ancestral (Supplementary Fig. 5) and BA.4/5 peptides (Fig. 9). Therefore, the subsequent analysis represents the response to BA.4/5 peptide stimulation.

The uniform manifold approximation and projection (UMAP) revealed significant differences in S-specific CD4^+^ T-cell phenotypes, especially between the transplant recipient groups and pHCs (Fig. 9a). The unsupervised clustering algorithm that uses k-nearest neighbors density estimation, Xshift^35^, was then applied, and identified eight distinct S-specific CD4^+^ T-cell clusters on the UMAP (Fig. 9b). The mean fluorescent intensities (MFIs) of markers expressed in each cluster are depicted in Fig. 9c. The frequencies of three clusters (6, 7, and 8) were statistically significantly different among the groups (Fig. 9d, e). Clusters 6 and 7 were enriched in the pHC group compared to the transplant recipient groups (Fig. 9d). Cells in these two clusters were metabolically active (GLUT1^+^, PD-1^+^), and expressed comparatively very high levels of IFN-γ (Fig. 9e). Cluster 7 cells were more polyfunctional than cluster 6 cells, expressing high frequencies of all four cytokines. Clusters 6 and 7 also expressed CD27 and CD28, consistent with a functional memory T-cell phenotype. Monovalently and bivalently vaccinated pSOTRs had the highest frequency of S-specific CD4^+^ T cells in cluster 8 (Fig. 9d, e). Cluster 8 cells expressed high levels of cytokines TNF and IL-2, but very little IFN-γ compared to other clusters (Fig. 9e). This is consistent with our polyfunctionality results in which pSOTR bivalent recipient CD4^+^ T cells co-expressed TNF and IL-2 (Fig. 8). Similar to clusters 6 and 7, cluster 8 cells expressed CD27, CD28, PD-1 and GLUT1, indicative of activated and functional memory T-cell phenotype.

**Fig. 10:**
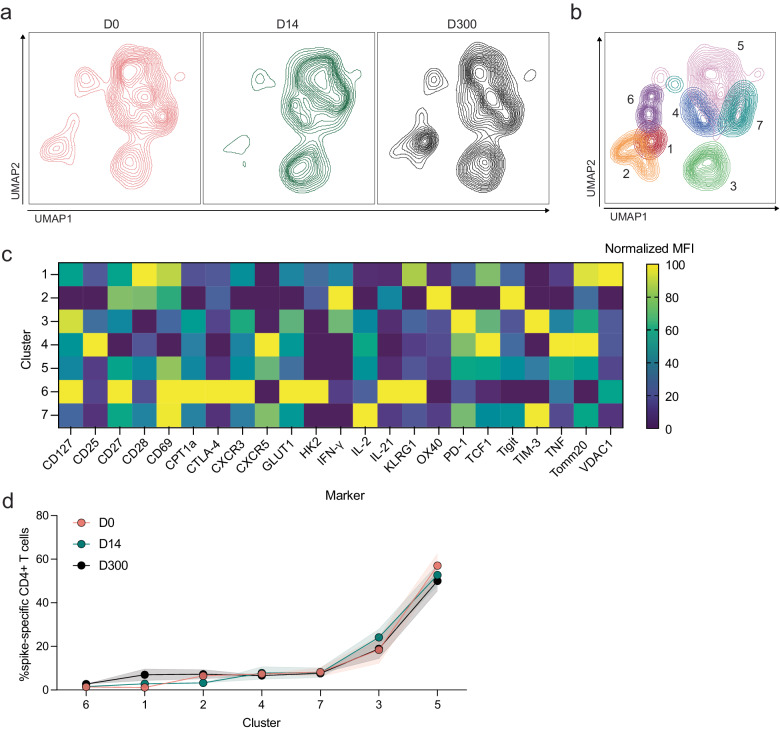
Bivalent boosting further promotes altered CD4^+^ T-cell phenotype in pSOTRs. **a** UMAP dimension reduction plot for each group. **b** Unsupervised clustering algorithm Xshift identified 7 clusters on the UMAP. **c** Heatmap of normalized mean fluorescent intensity (MFI) values of markers expressed in each cluster. **d** Frequency of clusters in each group. Two-way ANOVA with Tukey correction, all not significant. Error bars represent standard error of the mean.

We then applied the same analytical pipeline to cells from bivalent recipients prior to boosting, at peak and ten months post-vaccination. Again, no significant differences in S-specific CD4^+^ T-cell phenotypes were observed between responses to the ancestral (Supplementary Fig. 6) and BA.4/5 peptides (Fig. 10). The UMAP revealed slight differences in S-specific CD4^+^ T-cell phenotypes (Fig. 10a), especially expression of various cell markers on day 14 compared to days 0 and 300, and Xshift then identified seven S-specific CD4^+^ T-cell clusters on the UMAP (Fig. 10b). MFIs of markers expressed in each cluster are depicted in Fig. 10c. All seven clusters were present in comparable frequencies in each group (Fig. 10d).

Overall, our analysis of S-specific CD4^+^ T cells induced in response to BA.4/5 peptide stimulation suggests that despite immunosuppression, pSOTR recipients can generate metabolically active S-specific CD4^+^ T cells that are qualitatively distinct, primarily producing TNF and IL-2, less IL-21 and very little IFN-γ relative to pHCs. T cells of this phenotype were enhanced following bivalent vaccination, demonstrating that bivalent vaccination did not result in a higher proportion of T cells producing IFN-γ. This is distinct from CD4^+^ T cells generated in pHCs that were also metabolically active but mostly produced IFN-γ. Since monovalent pSOTR recipients produce similar frequencies of IFN-γ-producing CD4^+^ T cells as bivalent recipients, we hypothesize that immunosuppressive regimens alter the S-specific CD4^+^ T-cell compartment rather than that IFN-γ production is associated with fewer vaccine doses.

### Spike-specific CD4^+^ T-cell proliferation correlates with cytokine production in healthy children but not pSOTRs

**Fig. 11:**
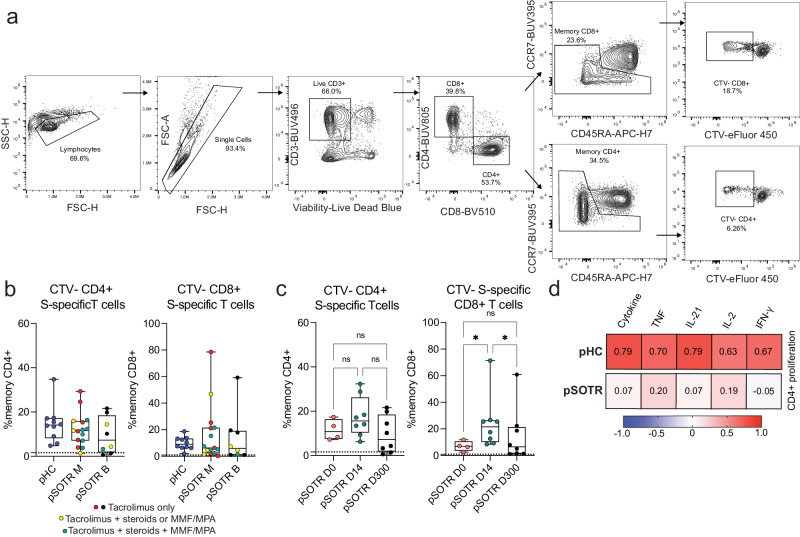
S-specific T-cell proliferation in response to ancestral spike peptides in pHCs and pSOTRs. **a** Representative gating of cell trace violet-labeled, S-specific, proliferating daughter T cells in response to ancestral SARS-CoV-2 S peptides. **b** Proliferating S-specific CD4^+^ and CD8^+^ T cells (% memory) in pHC, pSOTR M, and pSOTR B groups at the time of waning immunity. No significant relationships. Boxplots were used to summarize data (median, 1st–3rd quartiles (IRQ), whiskers represent minimum and maximum values). **c** Proliferating CD4^+^ and CD8^+^ T cells (% memory) in pSOTR B at days 0, 14, and 300. Kruskal-Wallis test, *p < 0.05, ns = not significant. Boxplots were used to summarize data (median, 1st–3rd quartiles (IRQ), whiskers represent minimum and maximum values). **d** Heatmap depicting correlations between proliferation of S-specific CD4^+^ T cells and cytokine production in pHCs and pSOTRs. The “cytokine” category combines TNF, IL-21, IL-2, and IFN-γ production. Pearson correlation coefficients are depicted in each square. Higher coefficient represents greater correlation.

Finally, we performed T-cell proliferation assay to further assess T-cell responses following vaccination (Fig. 11a). Cell trace violet dye-labeled PBMCs were cultured for five days in the presence of ancestral S peptides to drive the proliferation of S-specific T cells. Individuals who received bivalent vaccination exhibited the highest proliferation 14 days post-vaccination, as expected (Fig. 11b, c). Surprisingly, the pHC and pSOTR groups showed comparable S-specific T-cell proliferation at the time of waning immunity, potentially because children generally require lower doses of immunosuppressive regimes compared to adults. Interestingly, S-specific CD8^+^ T cells exhibited remarkable proliferation despite limited cytokine production. We then correlated CD4^+^ T cells proliferation with cytokine production and found a strong correlation between cytokine production and CD4^+^ T-cell proliferation in healthy children, but this correlation was not observed in pSOTRs (Fig. 11d). This further suggests that cytokine production and proliferation of S-specific CD4^+^ T cells is dysregulated in pSOTRs.

## Discussion

Adult SOTRs have impaired antibody titers, neutralizing capacity, and CD8^+^ T-cell responses following two- and three- doses of vaccination compared to healthy adults^1,2,26^. However, humoral and T-cell responses to mRNA COVID-19 vaccination have not been comprehensively evaluated in pSOTRs or healthy children. Therefore, we examined pSOTR and pHC responses to both vaccination and vaccination plus infection by analyzing total anti-SARS-CoV-2 IgG titers, surrogate neutralization capacity, and cytokine production and proliferation of S-specific CD4^+^ and CD8^+^ T cells. Although no pHCs received the bivalent dose, they are an excellent comparison group, representative of the healthy pediatric population in the U.S., both in terms of number of vaccines received and previous history of COVID-19. Monovalently vaccinated pSOTRs received, on average one additional ancestral mRNA vaccine and demonstrated comparable antibody titers, surrogate neutralization capacity, cytokine production and proliferation of S-specific CD4^+^ T cells compared to pHCs. This is in contrast to greater IgG titers, improved surrogate neutralization, and increased cytokine production in response to S protein among pSOTRs at six months post-last vaccine dose compared to adult SOTRs at peak vaccine responses. Further, bivalent boosting in pSOTRs enhanced IgG titers, surrogate neutralization and S-specific CD4^+^ T-cell cytokine production and polyfunctionality two weeks post-vaccination, but limited CD8^+^ T-cell responses remained. pHCs also exhibited limited CD8^+^ T-cell responses, suggesting that mRNA vaccines do not elicit strong CD8^+^ T-cell responses in children, as had been documented in adults^26,30,36^. Interestingly, pSOTRs displayed a distinct S-specific CD4^+^ T-cell phenotype that was metabolically active and produced primarily IL-2 and TNF cytokines with a lower proportion of T cells making IFN-γ, and this phenotype was enhanced in bivalently boosted individuals. SARS-CoV-2 infection did not have significant impact on humoral and T-cell responses in our study, likely because responses to vaccination were robust. Overall, our results demonstrate pSOTRs can mount humoral responses comparable to their immunocompetent siblings but have altered CD4^+^ T-cell responses.

A prior study of the ancestral monovalent COVID-19 vaccines in immunosuppressed children with inflammatory bowel disease (pIBD) demonstrated no significant difference in anti-S IgG titers between pIBDs and pHCs, the only immunologic parameter analyzed^37^. Similarly, we found that although pHCs received, on average one fewer vaccine dose compared to pSOTRs, the two groups mounted comparable IgG titers and exhibited similar surrogate neutralization capacity. Bivalent pSOTR recipients exhibited considerably improved antibody responses at peak, suggesting that despite immunosuppression, pSOTR immune responses are successfully boosted by additional vaccine doses and/or the bivalent dose. However, not surprisingly, both humoral and T-cell responses waned over time to pre-bivalent levels, suggesting that pSOTRs may benefit from a regular COVID-19 vaccination regimen. Bivalent dose recipients exhibited excellent inhibition against not only the ancestral strain, but all other variants, including BA.1, BA.2.75, BA.4.6, BA.5, BF.7, BQ.1, BQ.1.1, and the more recently circulating XBB.1 whose sublineages have dominated globally. In contrast to a recent study in adult SOTRs, in which approximately 30% of bivalent recipients fell below the 25% ACE2 inhibition cutoff^38^, none of the pediatric bivalent recipients in our study exhibited surrogate neutralization below this threshold. Although there was a notable enhancement in BA.5 surrogate neutralization within the bivalent recipients, it remained significantly lower than ancestral strain surrogate neutralization. This contrasts with the T-cell responses, which were not significantly different between ancestral and BA.4/5 peptide stimulation. In summary, with one additional dose, pSOTRs are capable of mounting comparable humoral responses to their immunocompetent siblings with responses against the ancestral strain, Omicron BA.5, and other variants of concern tested significantly improved by bivalent boost.

Prior studies evaluating vaccine-induced immune responses in transplant recipients have reported limited CD8^+^ T-cell responses following COVID-19 vaccination^15,26,39,40^. However, mRNA vaccine-induced CD8^+^ T-cell responses have not been extensively characterized in children. We detected reduced CD8^+^ T-cell responses compared to CD4^+^ responses in pSOTRs, even following the bivalent vaccine. Cytokine production by S-specific CD8^+^ T cells was also low in pHCs following stimulation with either ancestral or BA.4/5 peptides. These results suggest that COVID-19 mRNA vaccines do not elicit robust CD8^+^ T-cell responses in children, consistent with studies in adults.

In contrast, cytokine production by CD4^+^ T cells in response to ancestral and BA.4/5 S peptides was detectable in all groups. S-specific CD4^+^ T cells in pHCs and pSOTRs produced comparable frequencies of cytokines in response to BA.4/5 and ancestral peptides. However, pHCs demonstrated a superior ability to generate IFN-γ-producing S-specific CD4^+^ T cells in response to vaccination compared to pSOTRs. Furthermore, cytokine production by CD4^+^ T cells in response to ancestral and BA.4/5 S peptides increased significantly following the bivalent dose, especially Th1 cytokines IL-2 and TNF, as well as IL-21 cytokine produced by peripheral T follicular helper (pT_FH_) cells. pT_FH_ cells are transiently found in peripheral blood following infection or vaccination and robustly correlate with antibody responses and produce IL-21^41,42^. Increased IL-21 production is consistent with a recent study evaluating T-cell responses in adult SOTRs that identified IL-21-producing pT_FH_ that expressed high PD-1 and CXCR5 and were primarily present in individuals who have received at least three mRNA COVID-19 doses^26^. While pSOTRs also generated IFN-γ, the dominant cytokine produced were TNF and IL-2. These results were confirmed by our polyfunctionality analysis and unbiased analysis of S-specific T cells. The production of S-specific CD4^+^ T cells producing IL-2 and TNF was enhanced in bivalent recipients rather than shifting production to IFN-γ as seen in pHCs. Additionally, we detected comparable S-specific CD4^+^ T-cell proliferation in pHCs and pSOTRs; however, S-specific CD4^+^ T-cell proliferation in pSOTRs did not correlate with cytokine production, further supporting dysregulation of the mRNA vaccine-induced CD4^+^ T-cell compartment in pSOTRs.

As discussed, pHCs had the strongest IFN-γ responses despite, on average receiving on average only 2.4 mRNA vaccine doses. This is consistent with studies in immunocompetent adults reporting induction of durable S-specific CD4^+^ T cells producing IFN-γ following mRNA COVID-19 vaccination^43,44^. T cells could play a crucial role in defense against SARS-CoV-2 variants that can evade neutralizing antibodies. However, pSOTRs did not mount robust CD8^+^ T-cell responses and exhibited S-specific CD4^+^ T cells with a qualitatively different phenotype compared to pHCs, even following the bivalent dose. COVID-19 vaccination in immunosuppressed children induced the production of cells that while metabolically active, produce different cytokines compared to immunocompetent individuals. It is unknown whether the TNF- and IL-2-producing T cells detected in pSOTRs are capable of protection during subsequent reinfection and future studies will need to evaluate this phenotype. Given that pSOTRs had substantially lower frequency of IFN-γ producing T cells and IFN-γ has been shown to induce antiviral interferon-stimulated genes and to suppress SARS-CoV-2 replication in a dose-dependent manner^45^, additional vaccine doses that boost humoral responses may be the best strategy for protecting this population.

This study has several limitations, including limited sample size, differences in the number of vaccines received, and variable COVID-19 histories. Additionally, we did not perform live virus neutralization but used a surrogate neutralization assay that has demonstrated a high degree of correlation with live virus neutralization in previous studies in adult SOTRs^23,24^. Despite these limitations, this study comprehensively evaluates antibody and T-cell responses to mRNA COVID-19 vaccines in children, including the mRNA bivalent vaccine.

In conclusion, our findings demonstrate that despite immunosuppression, pSOTRs are capable of mounting humoral and CD4 T^+^ cell responses to both the ancestral strain and new variants of concern after receiving the primary series and multiple booster doses of mRNA COVID-19 vaccines. Notably, pSOTRs mounted more robust humoral and CD4^+^ T-cell responses than adult SOTRs. However, pSOTR participants displayed a distinct S-specific CD4^+^ T-cell phenotype, which was metabolically active (GLUT1^+^ PD-1^+^) but produced different cytokines (TNF, IL-2) compared to pHCs. The extent to which this phenotype offers protection against severe disease requires further investigation and supports the need to boost humoral responses in this population. Overall, these data provide insight into post-vaccine COVID-19 T-cell and antibody responses in pSOTRs to facilitate the selection of the optimal vaccine regimen in this patient population.

## Methods

### Recruitment and study approval

A total of 20 pSOTR monovalent mRNA vaccine recipients, nine pSOTR bivalent recipients, and ten pHCs (siblings of pSOTR participants) were enrolled in a national prospective, observational cohort (Johns Hopkins University IRB00248540) as previously described^2,46^. All pSOTR participants were recruited virtually, and their legal guardians provided detailed transplant history as well as oral informed consent (waiver of written consent granted). All vaccines were administered independently in the community without study team input. All pSOTR participants have received either kidney, liver, or heart transplants (Supplementary Table 1). Monovalently vaccinated pSOTR and pHC participants received, on average 3.4 and 2.4 mRNA vaccine doses, respectively (all Pfizer-BioNTech BNT162b2). Bivalent vaccine recipients received, on average 5.0 vaccine doses, including the bivalent dose (Pfizer-BioNTech COVID-19 Vaccine, Bivalent (Original and Omicron BA.4/BA.5) or SVAX Bivalent Original/Omicron BA.4-5). Three individuals are represented in both the monovalent and bivalent pSOTR group as we obtained PBMC samples for these participants before and after the mRNA bivalent booster dose. Additionally, approximately half of participants had a documented history of COVID-19, but anti-N titers were not always accurate in determining previous exposure (Fig. 1b). Hence, we relied primarily on self-reported and documented information regarding COVID-19 history. pSOTRs and pHCs in the monovalent groups and bivalent pSOTRs had blood drawn approximately six months since the last vaccine dose. We also had samples from peak responses to the bivalent dose (Supplementary Table 1). Additionally, 38 adult SOTR participants in this study (Figs. 1f–h, 3e) were recruited as a part of the “COVID-19 antibody testing of recipients of solid organ transplants and patients with chronic diseases,” as previously described (Johns Hopkins University IRB00248540)^2,24,26,46^. Humoral responses were included for 38 adult individuals and cellular responses for a subset for which we had PBMC samples (19 individuals). All adult participants had not had COVID-19 and received three doses of ancestral mRNA COVID-19 vaccines (Pfizer-BioNTech BNT162b2 or Moderna mRNA-1273) and their demographic characteristics are included in Supplementary Table 4.

### Peripheral blood mononuclear cell preparation

Blood was collected in acid-citrate dextrose tubes, and plasma was isolated by centrifugation and stored at –80°C until further analysis. PBMCs were isolated within 24 h of blood collection, as previously described^47^. Aliquots of PBMCs were stored in liquid nitrogen freezers until further analysis.

### Anti-SARS-CoV-2 IgG titers

Plasma was thawed and SARS-CoV-2 anti-N, anti-RBD, and anti-S IgG were measured using the multiplex chemiluminescent MesoScale Diagnostics (MSD) V-PLEX COVID-19 Respiratory Panel 3 Kit according to the manufacturer’s protocol at a dilution of 1:5000. Plates were read on MSD QuickPlex SQ 120, and arbitrary units were calculated using MSD Discovery Workbench software according to the manufacturer’s protocol. Conversion to WHO binding antibody units per milliliter was done by multiplying by the manufacturer’s recommended conversion factor. The positivity cutoff was determined by the manufacturer based on pre-pandemic serum samples and PCR-confirmed cases during the pre–SARS-CoV-2 vaccine period of the pandemic.

### ACE2 inhibition surrogate neutralization assay

The MSD ACE2 inhibition assay was used to measure the inhibition of ACE2 receptor binding to the S protein (% ACE2 inhibition) as previously described^24^. All samples were assayed on MSD V-PLEX SARS-CoV-2 panels 27 and 34 at a dilution of 1:100. Plates were read on MSD QuickPlex SQ 120, and percentage inhibition was calculated using the manufacturer’s protocol.

### Antigen recall assay

An antigen recall assay was utilized to assess S antigen-specific T-cell cytokine production following in vitro restimulation. Participant PBMCs were thawed using a CryoThaw adaptor^48^ (Medax) into 10 mL of RPMI (Gibco) supplemented with 10% fetal bovine serum (FBS) (Atlanta Biologicals). PBMCs were rested for approximately 6 h following thaw. Subsequently, 1e6 cells were cultured in 200 mL of RPMI supplemented with 10% FBS in a 96 well plate and stimulated with 1 mg/mL ancestral (PM-WCPV-S-1) or Omicron BA.4/5 (PM-SARS2-SMUT10-1) SARS-CoV-2 S peptide pools (JPT Peptide Technologies) that have been resuspended in dimethyl sulfoxide (DMSO) in presence of 10 mg/mL of brefeldin A (Millipore Sigma) overnight (approximately 14 h). Each pool is comprised of 315 15mers with an 11-amino acid overlap, spanning the entire SARS-CoV-2 spike protein. The BA.4/5 peptides encompass specific mutations of SARS-CoV-2, including T19I, L24del, P25del, P26del, A27S, H69del, V70del, G142D, V213G, G339D, S371F, S373P, S375F, T376A, D405N, R408S, K417N, N440K, L452R, S477N, T478K, E484A, F486V, Q498R, N501Y, Y505H, D614G, H655Y, N679K, P681H, N764K, D796Y, Q954H, N969K. Unstimulated wells were supplemented with equivalent volume DMSO and brefeldin A for all samples. The following day, surface and intracellular staining was performed for flow cytometry. All samples had individual unstimulated conditions (DMSO only) and stimulated conditions (ancestral or BA.5) and were all background subtracted. If a sample was negative or 0, it was changed to the lowest detectable value on the day the samples were run. These were considered a nonresponse. Peptides were prescreened for background activity in pre-pandemic samples and determined to have background activity comparable to DMSO-only controls. Antigen recall assays were performed twice, once with pSOTR M, pHC and adult SOTR PBMC samples, and subsequently repeated with pSOTR M, pSOTR B, and pHC PBMC samples.

### Flow cytometry staining

Cells were washed once in PBS and immediately stained for viability with Live/Dead Fixable Blue Dead Cell Stain (Invitrogen, L34962) for 10 min at room temperature. Cell surface staining was performed in 50 μL of 20% BD Horizon Brilliant Stain Buffer + PBS with surface stain Ab cocktail for 20 min at room temperature. The following antibodies were used for surface staining: anti-human CD4 (BD Biosciences, 612887, 1:80 dilution), CD8α (BioLegend, 300934, 1:635), CD45RA (BD Biosciences, 560674, 1:160 dilution), CCR7 (BD Biosciences, 749655, 1:20 dilution), CD25 (BD Biosciences, 612918, 1:40 dilution), CD27 (BD Biosciences, 563327, 1:320 dilution), CD28 (BD Biosciences, custom, 1:40 dilution), CD69 (BD Biosciences, 562989, 1:25 dilution), CD127 (BD Biosciences, custom, 1:80 dilution), CXCR3 (BD Biosciences, 740603, 1:20 dilution), CXCR5 (BD Biosciences, custom, 1:2500 dilution), -KLRG1 (BD Biosciences, 565393, 1:40 dilution), PD-1 (BD Biosciences, 750260, 1:40 dilution), TIM-3 (BD Biosciences, 748820, 1:40 dilution), Tigit (BD Biosciences, custom, 1:320 dilution), OX40 (CD134) (BD Biosciences, 563664, 1:320 dilution), and CTLA-4 (CD152) (BD Biosciences, custom, 1:635 dilution). Cells were fixed and permeabilized with eBioscience FoxP3/Transcription Factor Staining Buffer Set with 1× Fixation/Permeabilization reagent for 20 min at room temperature. Cells were washed with 1× Permeabilization/Wash buffer. Intracellular staining (ICS) was performed in 50 μL 1× Permeabilization/Wash buffer with ICS Ab cocktail for 20 min at room temperature using the following antibodies: anti-human IFN-γ (BD Biosciences, 566394, 1:1250 dilution), TNF (BD Biosciences, 566359, 1:160 dilution), IL-2 (BD Biosciences, 564164, 1:80 dilution), IL-21 (BioLegend, 513004, 1:50 dilution), HK2 (Abcam, ab209847, 1:80 dilution), CPT1a (Abcam, ab128568, 1:635 dilution), Tomm20 (Abcam, ab210047, 1:500 dilution), GLUT1 (Abcam, ab195020, 1:500 dilution), VDAC1 (Abcam, ab14734, 1:80 dilution), TCF1 (R&D Systems, IC8224G, 1:80 dilution), and CD3 (BD Biosciences, 612940, 1:800 dilution). Cells were washed once with Permeabilization/Wash buffer, then resuspended in 1% paraformaldehyde. Samples were run on a 4-laser (16UV-16V-15B-8R) Cytek Biosciences Aurora spectral flow cytometer and collected using the SpectoFlo v3.1 software. Additional information on flow cytometry antibodies used for phenotypic and metabolic analyses can be found in Supplementary Table 3.

### Flow cytometry analysis

FCS files were analyzed using FlowJo v10.9.0 software using manual gating and plugins for UMAP (v3.3.3), Xshift (v1.4.1), AutoGateCategorical (v2.6.1) and ClusterExplorer (v1.7.6). Frequencies of clusters identified by Xshift were applied to individual samples, and the frequency of the total population was determined. These frequencies were then stratified according to the indicated parameter and analyzed in GraphPad Prism v9 and v10. Polyfunctional T-cell responses were analyzed using Pestle v2.0 and SPICE 6 v6.1.

### T-cell proliferation assay

Participant PBMCs were thawed using a CryoThaw adaptor^48^ into 10 mL of RPMI supplemented with 10% FBS. PBMCs were rested for approximately 4 h following thaw. Subsequently, 1e6 cells were labeled with cell trace violet fluorescent dye following the manufacturer’s instructions (Thermo Fisher) and cultured in 1 mL of RPMI supplemented with 10% FBS in FACS tubes and stimulated for five days with 1 mg/mL ancestral peptides (JPT Peptide Technologies) that have been resuspended in DMSO. Unstimulated samples were supplemented with equivalent volume DMSO. After five days, surface staining was performed for flow cytometry using Invitrogen Live/Dead Fixable Blue Dead Cell Stain and anti-human CD3 (BUV661, BD Biosciences), CD4 (BUV805, BD Biosciences), CD8α (BV510, BioLegend), CCR7 (BUV395, BD Biosciences) and CD45RA (APC-H7, BD Biosciences) antibodies. T-cell proliferation assays were performed on participants for which we had sufficient PBMC samples.

### Statistical analyses

Statistical analyses were conducted using GraphPad Prism v9 and v10 and SPICE 6 v6.1. Data are presented as mean *±* standard error of the mean (SEM) unless otherwise indicated. Figure legends specify statistical tests used. A significance level of less than 0.05 (2-sided P value) was deemed statistically significant. Several sensitivity analyses were employed to assess the robustness of findings in comparing pSOTRs versus pHC immune responses. Specifically, multivariable linear regression analysis was conducted to examine T-cell cytokine production and antibody responses (Supplementary Table 2). We assessed the linearity assumption through a QQ plot and collinearity using the variance inflation factor. First, we used three crude models for the outcomes of S-specific CD4^+^ and CD8^+^ T-cell cytokine production, total IgG titers, and surrogate neutralization capacity. Differences between pSOTRs and pHCs were first assessed after adjusting for prior COVID-19, mycophenolate mofetil (MMF) use, and liver transplant history. The robustness of findings was further assessed in an expanded model also adjusting for age, the number of vaccines received before blood collection, and the time between vaccination and sample collection. Results and inferences were similar among the crude and adjusted models. In summary, our study employed several regression analyses to examine the immune responses in pSOTRs and pHCs, considering potential confounding factors to ensure the reliability of our results and none of these factors were found to significantly influence the primary results.

### Reporting summary

Further information on research design is available in the Nature Research Reporting Summary linked to this article.

### Supplementary information

Supplemental Information

REPORTING SUMMARY

## Data Availability

Deidentified data supporting the findings of this study are available from the corresponding authors upon request.
