# Supplementary material for: COVID-19 vaccination induces distinct T-cell responses in pediatric solid organ transplant recipients and immunocompetent children
Source: NPJ Vaccines. 2024 Apr 5;9:73. doi: 10.1038/s41541-024-00866-4 (PMC10997632; doi:10.1038/s41541-024-00866-4)
Supplement: Supplementary file 1 — Supplemental Information [file 41541_2024_866_MOESM1_ESM.pdf]

COVID-19 vaccination induces distinct T-cell responses in pediatric solid organ transplant recipients and immunocompetent children

Katerina Roznik, Jiashu Xue, Georgia Stavrakis, T. Scott Johnston, Divya Kalluri, Rivka Ohsie, Caroline X. Qin, John McAteer, Dorry L. Segev, Douglas Mogul, William A. Werbel, Andrew H. Karaba, Elizabeth A. Thompson, and Andrea L. Cox

SUPPLEMENTARY INFORMATION

SUPPLEMENTARY FIGURES

Supplementary Fig. 1: Humoral responses in vaccinated vs. vaccinated and infected individuals.

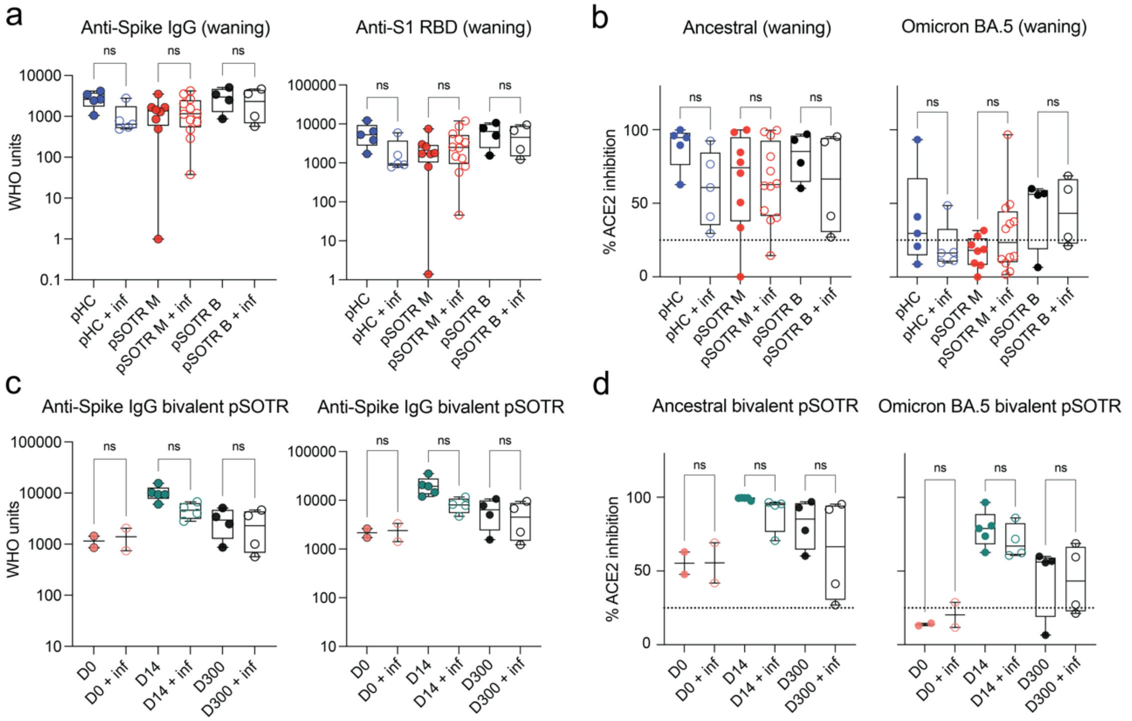

a, c Anti-Spike IgG and anti-receptor binding domain (RBD) IgG titers stratified by previous history of COVID-19 (+ inf = vaccination and natural infection). Kruskal-Wallis test with Dunn's correction, ns = not significant. Boxplots were used to summarize data (median, 1st–3rd quartiles (IRQ), whiskers represent minimum and maximum values). **b, d** Ancestral strain and Omicron BA.5 ACE2 binding inhibition stratified by previous history of COVID-19 (+ inf = vaccination and natural infection). Kruskal-Wallis tests with Dunn's correction, ns = not significant. Boxplots were used to summarize data (median, 1st–3rd quartiles (IRQ), whiskers represent minimum and maximum values).

Supplementary Fig. 2: Spike-specific CD4^{+} T cell responses in vaccinated vs. vaccinated and infected individuals.

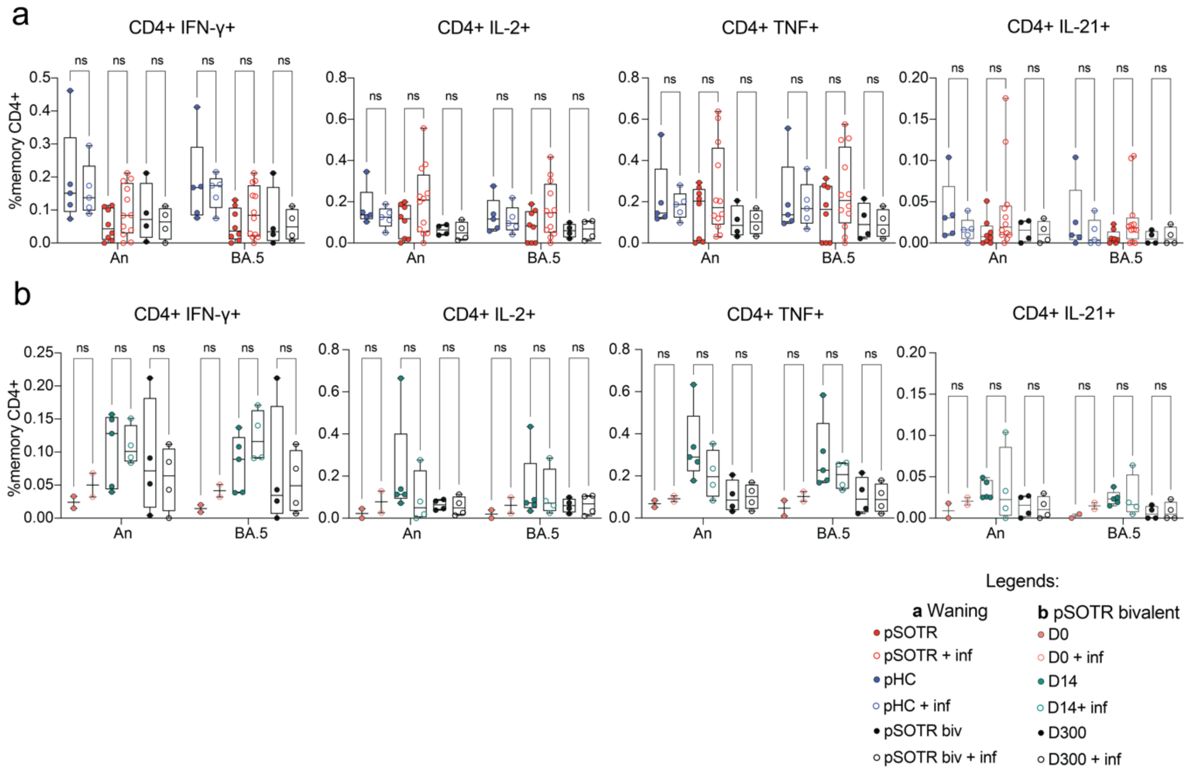

a, b Cytokine production by spike antigen-specific CD4^{+} T cells in response to ancestral or BA.4/5 S peptide stimulation stratified by previous history of COVID-19 (+ inf = vaccination and natural infection). Two-way ANOVA with Tukey correction, ns = not significant. Boxplots were used to summarize data (median, 1st–3rd quartiles (IRQ), whiskers represent minimum and maximum values).

Supplementary Fig. 3: Spike-specific CD8⁺ T cell responses in vaccinated vs. vaccinated and infected individuals.

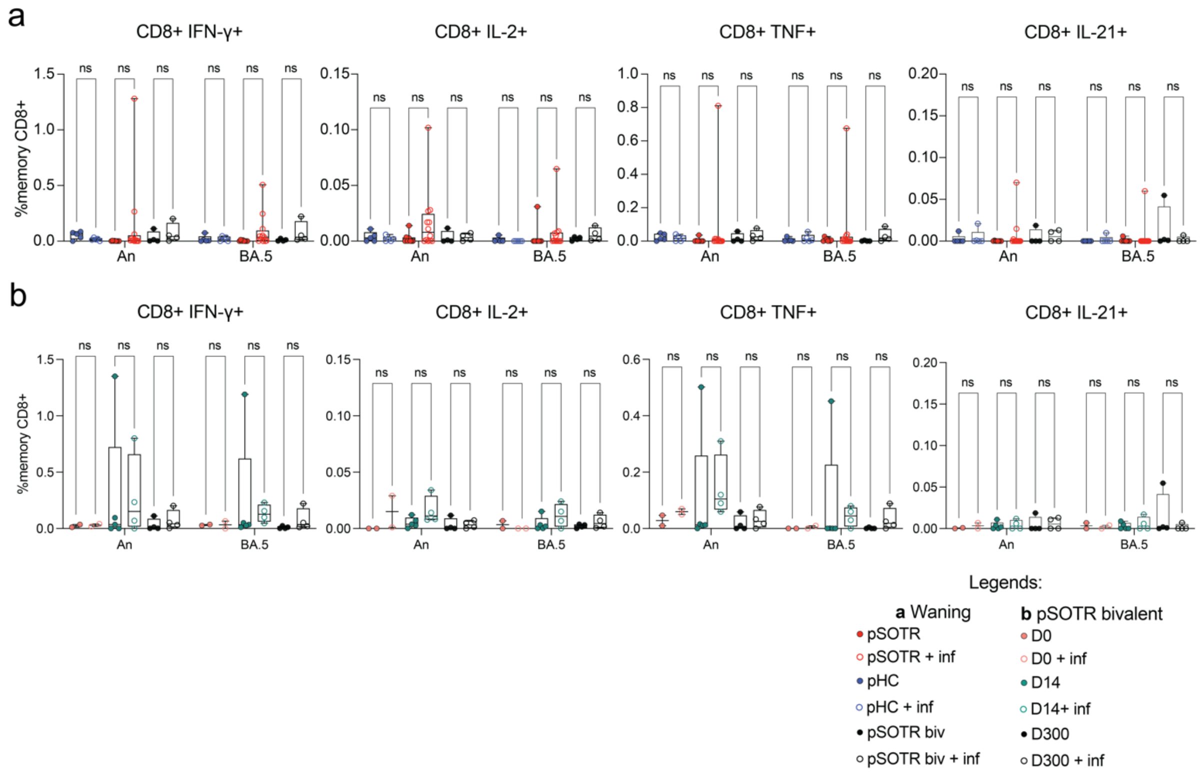

a, b Cytokine production by spike antigen-specific CD8⁺ T cells in response to ancestral or BA.4/5 S peptide stimulation stratified by previous history of COVID-19 (+ inf = vaccination and natural infection). Two-way ANOVA with Tukey correction, ns = not significant. Boxplots were used to summarize data (median, 1st–3rd quartiles (IRQ), whiskers represent minimum and maximum values).

Supplementary Fig. 4: Polyfunctionality of spike-specific CD4$^+$ T cells in bivalent recipients stratified by COVID-19 history.

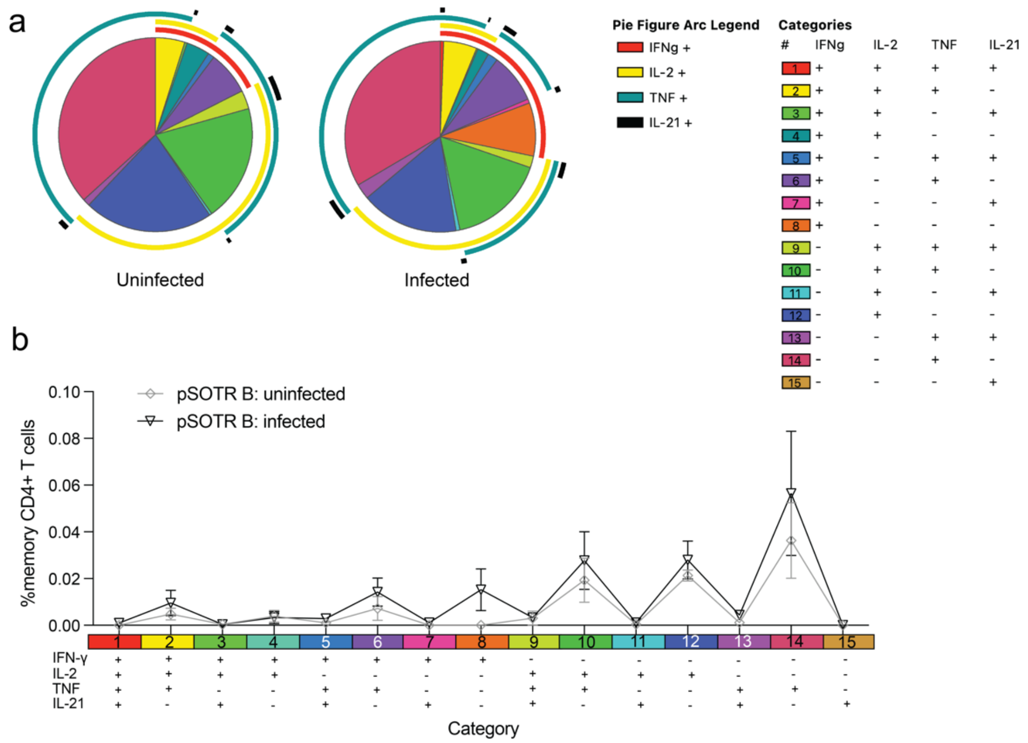

a Pie charts of polyfunctional responses in response to ancestral or BA.4/5 S peptide stimulation stratified by previous history of COVID-19 in pSOTRs with waning immune responses. Arcs identify slices of the pie that express each specific cytokine. **b** Frequencies of CD4$^+$ T cells producing cytokine combinations in response to ancestral and BA.4/5 peptide stimulation. Two-way ANOVA with Tukey correction, no statistically significant differences. Error bars represent standard error of the mean.

Supplementary Fig. 5: Phenotypic evaluation of S-specific CD4+ T cells in response to ancestral S peptides.

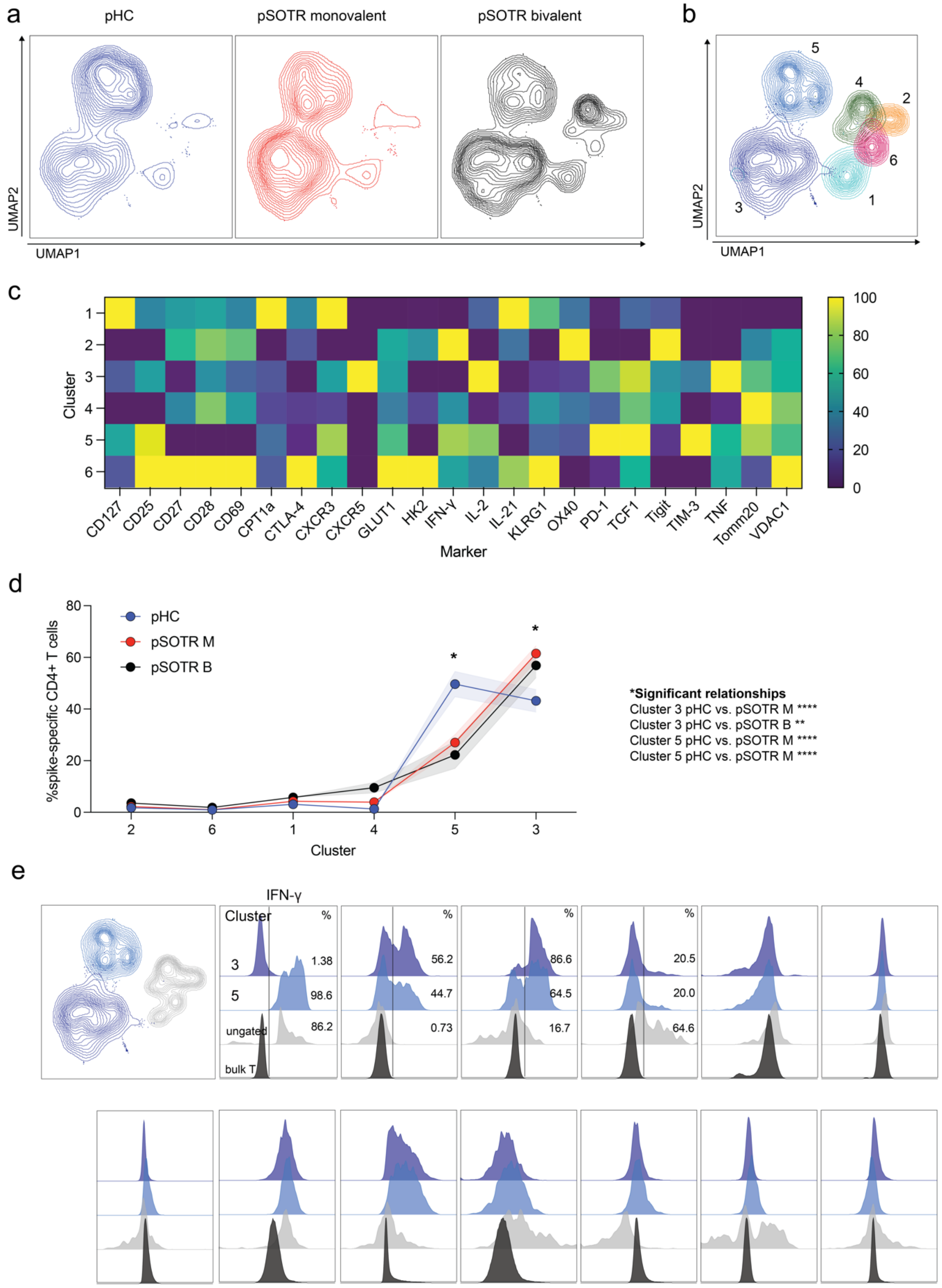

a UMAP dimension reduction plot for each group. **b** Xshift algorithm identified 6 clusters on the UMAP. **c** Heatmap of normalized mean fluorescent intensity (MFI) values of markers expressed in each cluster. **d** Frequency of clusters in each group. Two-way ANOVA with Tukey correction, *p<0.05, **p<0.01, ***p<0.001, ****p<0.0001. Error bars represent standard error of the mean. **e** MFI plots for significant clusters determined in panel **d**, ungated clusters (gray) and bulk T cells (black).

Supplementary Fig. 6: Phenotypic evaluation of S-specific CD4$^+$ T cells in response to ancestral S peptides in bivalent vaccine recipients.

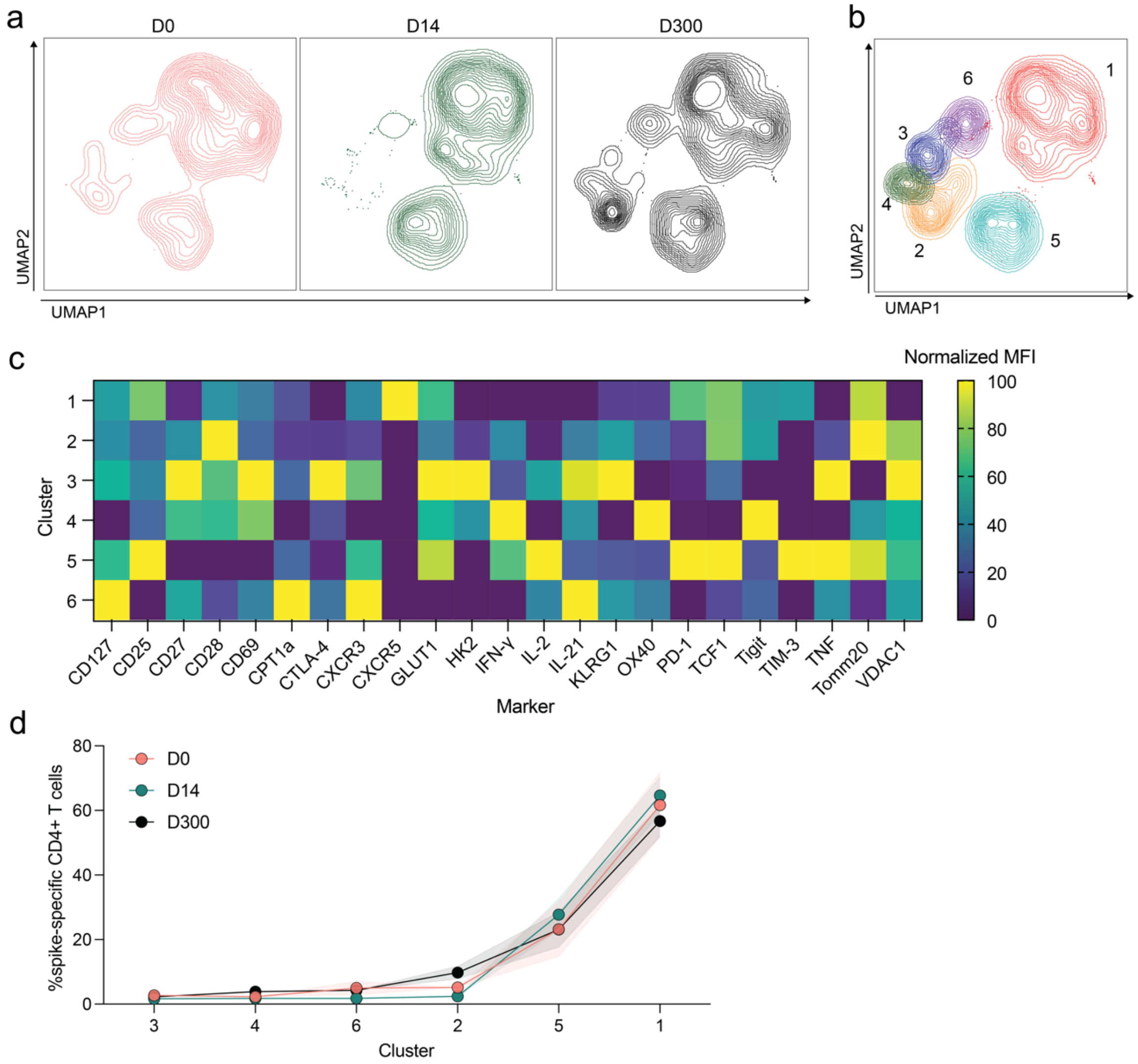

a UMAP dimension reduction plot for each group. **b** Xshift algorithm identified 6 clusters on the UMAP. **c** Heatmap of normalized mean fluorescent intensity (MFI) values of markers expressed in each cluster. **d** Frequency of clusters in each group. Two-way ANOVA with Tukey correction, not significant relationships. Error bars represent standard error of the mean.

SUPPLEMENTARY TABLES

Supplementary Table 1. Demographic characteristics of the pediatric cohort.

	**pSOTR monovalent waning**	**pHC monovalent waning**	**pSOTR bivalent waning**	**pSOTR bivalent day 14**
**Sample size (n)**	20	10	8*	9
**Demographics**				
Age (median)	13.5 (10, 14.25)	12 (11.25, 13.75)	14.0 (12, 15)	
Female, n (%)	10 (50)	5 (50)	4 (44)	
White, n (%)	12 (60)	5 (50)	7 (78)	
Hispanic, n (%)	1 (5)	1 (10)	1 (11)	
**# Vaccines at draw**				
2	1 (5)	6 (60)	/	
3	10 (50)	4 (40)	/	
4	8 (40)	/	1 (11)	
5	1 (5)	/	7 (78)	
6	/	/	1 (11)	
Mean	3.45	2.40	5	
Median	3	2	5	
**Vaccine to draw in days, median**	155 (123.5, 266.8)	183 (145, 194)	294 (285, 299)	14.0
**COVID-19 history (yes), n (%)**	11 (55)	5 (50)	4 (44)	4 (44)
**Time since COVID-19, median**	138 (104.5, 265.5)	140 (73, 294.5)	226 (131.5, 311)	
**TX organ, n (%)**				
Kidney	7 (35)	/	4 (44)	
Liver	9 (45)	/	2 (22)	
Heart	4 (20)	/	3 (33)	
**Time since TX in yrs, median**	7 (3.75, 10)	/	9 (5, 10)	
**Immunosuppressive regimen, n (%)**				
Antimetabolite (MMF)	8 (40)	/	3 (33)	
Corticosteroids	9 (45)	/	2 (22)	
mTOR inhibitors	5 (25)	/	3 (33)	
Tacrolimus	20 (100)	/	5 (56)	

*One individual from the pSOTR bivalent day 14 group was lost to follow-up.

Supplementary Table 2. Humoral and CD4^{+} T cell responses adjusted for several demographic variables.

T cell response	pSOTR (n=20) vs. healthy sibling control (n=10)				
	Crude β1	Crude β2	Crude β3	Adjusted β[a]	P-value
CD4_IFNg_BA.5	-0.093	-0.100	-0.097	-0.094	0.002**
CD4_IL2_BA.5	0.003	-0.011	0.014	0.005	0.892
CD4_IL21_BA.5	-0.001	-0.012	0.001	-1.63e-04	0.989
CD4_TNF_BA.5	0.004	-0.024	0.002	0.006	0.928
CD4_IFNg_An	-0.099	-0.107	-0.103	-0.100	0.004**
CD4_IL2_An	0.011	-0.013	0.026	0.012	0.798
CD4_IL21_An	0.002	-0.013	0.0002	2.69e-03	0.867
CD4_TNF_An	0.007	-0.012	0.0054	0.008	0.904
Anti-S IgG	-370.2	-19.72	-358.7	-430.3	0.382
An %ACE2 inhibition	-10.53	-5.119	-13.54	-11.76	0.265
BA.5 %ACE2 inhibition	-5.344	1.192	-1.120	-5.682	0.526

Notes:

β1 was adjusted for history of COVID-19

β2 was adjusted for mycophenolate mofetil intake (MMF) vs. no MMF intake

β3 was adjusted for liver transplant history vs. no liver transplant history

[a] Adjusted for vaccine to blood draw time (days), age, number of vaccines prior to blood sample collection

An = ancestral, BA.5 = Omicron BA.5

Supplementary Table 3. Flow cytometry panel to characterize spike antigen-specific T cells responses.

Surface markers

Fluorophore	Marker	Clone	Vendor	Catalog #
Live/Dead Blue	Viability	*NA*	Invitrogen	L34962
BUV805	CD4	SK3	BD Biosciences	612887
BV510	CD8α	HIT8a	BioLegend	300934
APC-H7	CD45RA	HI100	BD Biosciences	560674
BUV395	CCR7	2-L1-A	BD Biosciences	749655
BUV563	CD25	2A3	BD Biosciences	612918
BV786	CD27	L128	BD Biosciences	563327
BV570	CD28	RF8B2	BD Biosciences	Custom
BV605	CD69	FN50	BD Biosciences	562989
PE-Cy5	CD127	HIL-7R-M21	BD Biosciences	Custom
BV650	CXCR3	1C6	BD Biosciences	740603
BB790	CXCR5	B27	BD Biosciences	Custom
PE-CF594	KLRG1	2F1	BD Biosciences	565393

BUV661	PD-1 (CD279)	EH12.1	BD Biosciences	750260
BUV737	TIM-3	7D3	BD Biosciences	748820
BB660	Tigit	741182	BD Biosciences	Custom
BV711	OX40 (CD134)	ACT35	BD Biosciences	563664
PE-Cy7	CTLA-4 (CD152)	BNI3	BD Biosciences	Custom

Intracellular markers

**Fluorophore**	**Marker**	**Clone**	**Vendor**	**Catalog #**
BB700	IFN-γ	B27	BD Biosciences	566394
BV750	TNF	MAb11	BD Biosciences	566359
BV421	IL-2	MQ1-17H12	BD Biosciences	564164
PE	IL-21	3A3-N2	BioLegend	513004
Alexa Fluor 680	Hexokinase 2*	EPR20839	Abcam	ab209847
PE-Cy5.5	CPT1a*	8F6AE9	Abcam	ab128568
Alexa Fluor 405	Tomm20	EPR15581-54	Abcam	ab210047
Alexa Fluor 647	GLUT1	EPR3915	Abcam	ab195020
Alexa Fluor 532	VDAC1**	20B12AF2	Abcam	ab14734
Alexa Fluor 488	TCF1	812145	R&D Systems	IC8224G
BUV496	CD3	UCHT-1	BD Biosciences	612940

* Self-conjugated using Abcam Lighting Link Conjugation kits (ab201804, ab102899)
** Self-conjugated using Thermo Fisher Alexa Fluor Antibody Labeling kit (A20182)

Supplementary Table 4. Demographic characteristics of the adult cohort.

**Sample size (n)**	38
**Demographics**	
Age (median, IRQ)	55.8 (44, 67.5)
Female, n (%)	16 (42.1)
White, n (%)	31 (81.6)
**# Vaccines at draw, type**	
3 doses	38 (100)
Moderna	23 (60.5)
Pfizer/BNT	15 (39.5)
**COVID-19 history (yes), n (%)**	0 (0)
**TX organ, n (%)**	
Kidney	26 (68.4)
**Time since TX in yrs** (median, IRQ)	3.5 (2.1, 7.7)
**Immunosuppressive regimen, n (%)**	
Antimetabolite	28 (73.7)

Prednisone	18 (47.4)
Cyclosporine	1 (2.6)
Tacrolimus	33 (86.8)
Triple regimen	12 (31.6)
