# Supplementary material for: COVID-19 vaccination induces distinct T-cell responses in pediatric solid organ transplant recipients and immunocompetent children
Source: NPJ Vaccines. 2024 Apr 5;9:73. doi: 10.1038/s41541-024-00866-4 (PMC10997632; doi:10.1038/s41541-024-00866-4)
Supplement: Supplementary file 2 — REPORTING SUMMARY [file 41541_2024_866_MOESM2_ESM.pdf]

nature portfolio

Corresponding author(s): Andrea L. Cox

Last updated by author(s): Mar 4, 2024

Reporting Summary

Nature Portfolio wishes to improve the reproducibility of the work that we publish. This form provides structure for consistency and transparency in reporting. For further information on Nature Portfolio policies, see our Editorial Policies and the Editorial Policy Checklist.

Statistics

For all statistical analyses, confirm that the following items are present in the figure legend, table legend, main text, or Methods section.

n/a	Confirmed	
☐	☒	The exact sample size (n) for each experimental group/condition, given as a discrete number and unit of measurement
☐	☒	A statement on whether measurements were taken from distinct samples or whether the same sample was measured repeatedly
☐	☒	The statistical test(s) used AND whether they are one- or two-sided<br>*Only common tests should be described solely by name; describe more complex techniques in the Methods section.*
☐	☒	A description of all covariates tested
☐	☒	A description of any assumptions or corrections, such as tests of normality and adjustment for multiple comparisons
☐	☒	A full description of the statistical parameters including central tendency (e.g. means) or other basic estimates (e.g. regression coefficient) AND variation (e.g. standard deviation) or associated estimates of uncertainty (e.g. confidence intervals)
☐	☒	For null hypothesis testing, the test statistic (e.g. F, t, r) with confidence intervals, effect sizes, degrees of freedom and P value noted<br>*Give P values as exact values whenever suitable.*
☒	☐	For Bayesian analysis, information on the choice of priors and Markov chain Monte Carlo settings
☒	☐	For hierarchical and complex designs, identification of the appropriate level for tests and full reporting of outcomes
☒	☐	Estimates of effect sizes (e.g. Cohen's d, Pearson's r), indicating how they were calculated

Our web collection on statistics for biologists contains articles on many of the points above.

Software and code

Policy information about availability of computer code

Data collection	The software used to collect data was Cytek Biosciences' flow cytometry software SpectroFlo.
Data analysis	The following software was utilized to analyze data in this study: Pestle 2.0, SPICE 6, GraphPad Prism (versions 9 and 10), FlowJo (version 10.9.0).

For manuscripts utilizing custom algorithms or software that are central to the research but not yet described in published literature, software must be made available to editors and reviewers. We strongly encourage code deposition in a community repository (e.g. GitHub). See the Nature Portfolio guidelines for submitting code & software for further information.

Data

Policy information about availability of data

All manuscripts must include a data availability statement. This statement should provide the following information, where applicable:

- Accession codes, unique identifiers, or web links for publicly available datasets
- A description of any restrictions on data availability
- For clinical datasets or third party data, please ensure that the statement adheres to our policy

Deidentified data supporting the findings of this study are available from the corresponding authors upon request.

Research involving human participants, their data, or biological material

Policy information about studies with human participants or human data. See also policy information about sex, gender (identity/presentation), and sexual orientation and race, ethnicity and racism.

Reporting on sex and gender	Participant sex and gender information was collected at recruitment through self-reporting. Among the pediatric solid organ transplant recipients who received monovalent mRNA vaccines, 50% identified as female (n=10). For those who received the bivalent booster dose, 44% identified as female (n=4). In the healthy sibling group, 50% identified as female (n=5). Among adult solid organ transplant recipients in the study, 42% identified as female (n=16). Due to the absence of significant differences based on sex, we decided not to stratify results based on this criterion.
Reporting on race, ethnicity, or other socially relevant groupings	Data on participant race and ethnicity were gathered during recruitment through self-reporting. In the pediatric solid organ transplant recipient group receiving monovalent mRNA vaccines, twelve individuals identified as White (60%), and one individual (5%) reported Hispanic ethnicity. Among those in the pediatric solid organ transplant group who received the bivalent booster, seven individuals (78%) were White, and one (11%) reported Hispanic ethnicity. In the healthy sibling group, five individuals were White (50%), and one (10%) identified as Hispanic. Out of the 38 adult solid organ transplant recipients, 31 (81.6%) were White.
Population characteristics	Pediatric solid organ transplant recipients included in this study who received monovalent mRNA vaccines had a median age of 13.5 years (10, 14.25) and received a median of three (2, 4) monovalent ancestral mRNA COVID-19 vaccines. Seven (35%) individuals in this group received a kidney transplant. Eight individuals (40%) were receiving antimetabolite immunosuppressive drugs, nine (45%) corticosteroids, five mTOR inhibitors (25%), and 20 tacrolimus (100%) at the time of analysis. Pediatric solid organ transplant recipients who received the bivalent booster dose had a median age of 14 years (12, 15) and received a median of five (4, 6) mRNA COVID-19 vaccine doses. Four (44%) individuals in this group received a kidney transplant. Three (33%) individuals were on antimetabolite drugs, two (22%) received corticosteroids, three (33%) mTOR inhibitors, and five (56%) tacrolimus. Healthy siblings of pediatric solid organ transplant recipients were also recruited and their median age was 12 years (11.3, 13.8). The individuals in this study group received a median of two (2, 3) mRNA COVID-19 vaccine doses. Median age of the adult cohort was 55.8 years (44, 67.5) and every individual in this cohort received three doses of mRNA COVID-19 vaccines. The majority of this group received a kidney transplant (68.4%), and 28 individuals (73.7%) were reported to be on antimetabolite immunosuppressive medication, 18 (47.4%) on prednisone, 1 (2.6%) on cyclosporine, and 33 (86.8%) on tacrolimus. We conducted analysis further described in the Methods section to ensure that our results remained significant after accounting for immunosuppression (mycophenolate mofetil use), liver transplant history, age, number of vaccines received, and time between vaccination and sample collection. Additional population characteristics are provided in Supplementary Tables 1 (pediatric) and 4 (adult).
Recruitment	Pediatric solid organ transplant recipients, their siblings and the adult cohort were enrolled in a national prospective, observational cohort through a digital campaign. Pediatric solid organ transplant participants were recruited virtually, and their legal guardians provided detailed transplant history as well as oral informed consent (waiver of written consent granted). Adults in this study provided detailed transplant history as well as written informed consent.
Ethics oversight	This study was approved by the Johns Hopkins Institutional Review Board (IRB00248540).

Note that full information on the approval of the study protocol must also be provided in the manuscript.

Field-specific reporting

Please select the one below that is the best fit for your research. If you are not sure, read the appropriate sections before making your selection.

☒ Life sciences ☐ Behavioural & social sciences ☐ Ecological, evolutionary & environmental sciences

For a reference copy of the document with all sections, see nature.com/documents/nr-reporting-summary-flat.pdf

Life sciences study design

All studies must disclose on these points even when the disclosure is negative.

Sample size	Participants were recruited as part of a nationwide observational study. All pediatric individuals whose legal guardians agreed to provide blood samples were included. Due to the observational nature of the study, the pediatric sample size is limited, comprising 29 pediatric solid organ transplant recipients and 10 healthy sibling controls. The adult cohort consists of 38 individuals who received three mRNA vaccine doses and were never infected with SARS-CoV-2. The study included individuals from the adult cohort with available plasma and PBMC samples.
Data exclusions	No pediatric participants were excluded. A single adult participant was excluded based on suspicion of asymptomatic SARS-CoV-2 infection as this individual exhibited high anti-nucleocapsid antibody titers.
Replication	Initially, our experiments utilized PBMCs and plasma from monovalently vaccinated pediatric solid organ transplant recipients and their siblings. Subsequently, we acquired additional samples from pediatric solid organ transplant recipients who received the bivalent booster dose. All experiments were then repeated, incorporating both new and original samples, yielding consistent results. The data submitted in the original manuscript (August 2023) were from the initial experiments, whereas the resubmission (January 2024) included data from the repeated experiments.

Randomization	Allocation to study groups was not random. It was based on whether an individual had a transplanted organ or not (solid organ transplant recipient vs healthy control), whether an individual was an adult or a child, and based on the number of vaccines received (monovalent vs bivalent) and timing (baseline or day 0, peak antibody responses or day 14, waning responses).
Blinding	Not relevant to this study because all mRNA COVID-19 vaccines were administered independently in the community without study team input. Individuals in our study who are on immunosuppressive regimens were prescribed these medications by their physicians without study team input.

Reporting for specific materials, systems and methods

We require information from authors about some types of materials, experimental systems and methods used in many studies. Here, indicate whether each material, system or method listed is relevant to your study. If you are not sure if a list item applies to your research, read the appropriate section before selecting a response.

Materials & experimental systems

n/a	Involved in the study
☐	☒ Antibodies
☒	☐ Eukaryotic cell lines
☒	☐ Palaeontology and archaeology
☒	☐ Animals and other organisms
☒	☐ Clinical data
☒	☐ Dual use research of concern
☒	☐ Plants

Methods

n/a	Involved in the study
☒	☐ ChIP-seq
☐	☒ Flow cytometry
☒	☐ MRI-based neuroimaging

Antibodies

Antibodies used

We used the following surface stain antibodies: Anti-human CD4 (BUV805, clone SK3, BD Biosciences, catalog number 612887), CD8 (BV510, clone HIT8alpha, BioLegend, catalog number 300934), CD45RA (APC-H7, clone HI100, BD Biosciences, catalog number 560674), CCR7 (BUV395, clone 2-L1-A, BD Biosciences, catalog number 749655), CD25 (BUV563, clone 2A3, BD Biosciences, catalog number 612918), CD27 (BV786, clone L128, BD Biosciences, catalog number 563327), CD28 (BV570, clone RF8B2, BD Biosciences, catalog number 624298, custom conjugate), CD69 (BV605, clone FN50, BD Biosciences, catalog number 562989), CD127 (PE-Cy5, clone HIL-7R-M21, BD Biosciences, catalog number 624068, custom conjugate), CXCR3 (BV650, clone 1C6, BD Biosciences, catalog number 740603), CXCR5 (BB790, clone B27, BD Biosciences, catalog number 624296, custom conjugate), KLRG1 (PE-CF594, clone 2F1, BD Biosciences, catalog number 565393), PD-1 (BUV661, clone EH12.1, BD, Biosciences, catalog number 750260), TIM-3 (BUV737, clone 7D3, BD Biosciences, catalog number 748820), Tigit (BB660, clone 741182, BD Biosciences, catalog number 626349, custom conjugate), OX40 (BB660, clone ACT35, BD Biosciences, catalog number 563664), and CTLA-4 (PE-Cy7, clone BNI3, BD Biosciences, catalog number 624351, custom conjugate).

The following intracellular antibodies were used: IFN-gamma (BB700, clone B27, BD Biosciences, catalog number 566394), TNF (BV750, clone MAb11, BD Biosciences, catalog number 566359), IL-2 (BV421, clone MQ1-17H12, BD Biosciences, catalog number 564164), IL-21 (PE, clone 3A3-N2, BioLegend, catalog number 513004), Hexokinase 2 (Alexa Fluor 680, clone EPR29839, Abcam, catalog number ab228819), CPT1a (PE-Cy5.5, clone 8F6AE9, Abcam, catalog number ab128568), Tomm20 (Alexa Fluor 405, clone EPR15581-54, Abcam, catalog number ab210047), GLUT1 (Alexa Fluor 647, clone EPR3915, Abcam, catalog number ab195020), VDAC1 (Alexa Fluor 532, clone 20B12AF2, Abcam, catalog number ab14734), TCF1 (Alexa Fluor 488, clone 812145, R&D Systems, catalog number MAB8224), and CD3 (BUV496, clone UCHT1, BD Biosciences, catalog number 612940).

Additional information on flow cytometry antibodies used for phenotypic and metabolic analyses can be found in the Methods section and Supplementary Table 3.

Validation

All antibodies used in this manuscript are commercially available, including custom conjugates. Antibodies obtained from BioLegend and BD Biosciences were quality tested. According to BD Biosciences, the production process of their antibody conjugates undergoes stringent testing and validation to assure that it generates a high-quality conjugates with consistent performance and specific binding activity. Each lot of antibodies obtained from BioLegend is quality control tested by immunofluorescent staining with flow cytometric analysis. Additionally, the following antibodies were KO validated: VDAC1, CPT1a, and Hexokinase 2.

Plants

Seed stocks	n/a
Novel plant genotypes	*Describe the methods by which all novel plant genotypes were produced. This includes those generated by transgenic approaches, gene editing, chemical/radiation-based mutagenesis and hybridization. For transgenic lines, describe the transformation method, the number of independent lines analyzed and the generation upon which experiments were performed. For gene-edited lines, describe the editor used, the endogenous sequence targeted for editing, the targeting guide RNA sequence (if applicable) and how the editor was applied.*
Authentication	*Describe any authentication procedures for each seed stock used or novel genotype generated. Describe any experiments used to assess the effect of a mutation and, where applicable, how potential secondary effects (e.g. second site T-DNA insertions, mosiacism, off-target gene editing) were examined.*

Flow Cytometry

Plots

Confirm that:

☒ The axis labels state the marker and fluorochrome used (e.g. CD4-FITC).

☒ The axis scales are clearly visible. Include numbers along axes only for bottom left plot of group (a 'group' is an analysis of identical markers).

☒ All plots are contour plots with outliers or pseudocolor plots.

☒ A numerical value for number of cells or percentage (with statistics) is provided.

Methodology

Sample preparation	Participant PBMCs (pediatric and adult solid organ transplant recipients and healthy children) were obtained from blood samples. Blood was collected in acid citrate dextrose tubes, and plasma was isolated by centrifugation and stored at –80°C until further analysis. PBMCs were stored in liquid nitrogen until further analysis.<br><br>PBMC samples were thawd, rested for approximately 6 hours following thaw. Subsequently, 1e6 cells were cultured in 96 well plates and stimulated with ancestral or Omicron BA.4/5 SARS-CoV-2 S peptide pools in presence of brefeldin A overnight. Unstimulated wells were supplemented with equivalent volume DMSO and brefeldin A for all samples. The following day, surface and intracellular staining was performed for flow cytometry. All samples had individual unstimulated conditions (DMSO only) and stimulated conditions (ancestral or BA.5) and were all background subtracted. If a sample was negative or 0, it was changed to the lowest detectable value on the day the samples were run. These were considered a nonresponse. Peptides were prescreened for background activity in pre-pandemic samples and determined to have background activity comparable to DMSO-only controls.
Instrument	4-laser (16UV-16V-15B-8R) Cytek Biosciences Aurora spectral flow cytometer.
Software	To collect data, we used Cytek Biosciences' SpectroFlo v3.1 software. To analyze data, we used the FlowJo v10.9.0 software.
Cell population abundance	Our assays are designed to identify cytokine-producing SARS-CoV-2 spike-specific CD4 and CD8 T cells, which are not particularly abundant (see Figs. 3b and 5a). Each sample was divided into three conditions - unstimulated cells, and cells stimulated with ancestral spike peptides, and Omicron BA.4/5 spike peptides. We used our unstimulated conditions to set the gating strategies. We tested this protocol on healthy cells from prior to the COVID-19 pandemic and did not detect any spike-specific T cell populations (comparable to unstimulated condition). We also tested this method on healthy individuals who were infected with SARS-CoV-2 and vaccinated against SARS-CoV-2 to make sure we can identify and pull spike-specific CD4 and CD8 T cells.
Gating strategy	Gating strategies are detailed in our main figures (Fig. 3a, 3b, 5a, and 11a). To gate on SARS-CoV-2 spike-specific CD4 and CD8 T cells, we first gated on lymphocytes by size (FSC/SSC). We then gated on single cells, followed by CD3+ live cells (Q1 population) as live cells are negative for Live Dead Blue dye. We then distinguished between CD4 and CD8 T cells, followed by gating on CD4 and CD8 memory cells. To identify memory cells, we gated on CD45RA and CCR7 double negative populations (naive cells are CD45RA+CCR7+). We then visualized our spike-specific CD4 and CD8 T cells by selecting each cytokine on the y-axis (i.e., IFN-g, TNF, IL-2, and IL-21) vs. CD4/CD8 on the x-axis. We used unstimulated conditions (no spike peptides) to set our cytokine gates (see Fig. 3b and 5a).

☒ Tick this box to confirm that a figure exemplifying the gating strategy is provided in the Supplementary Information.
